# Multimodal learning of pheromone locations

**DOI:** 10.1096/fj.202100167R

**Published:** 2021-09-01

**Authors:** Meenakshi Pardasani, Shruti D. Marathe, Maitreyee Mandar Purnapatre, Urvashi Dalvi, Nixon M. Abraham

**Affiliations:** 1 Laboratory of Neural Circuits and Behaviour (LNCB), Department of Biology, Indian Institute of Science Education and Research (IISER), Pune, India; 2 Institute of Bioinformatics & Biotechnology, Savitribai Phule Pune University, Pune, India

**Keywords:** non-pheromonal volatiles discrimination, olfactory bulb, pheromone location preference, somatosensory cortex

## Abstract

Memorizing pheromonal locations is critical for many mammalian species as it involves finding mates and avoiding competitors. In rodents, pheromonal information is perceived by the main and accessory olfactory systems. However, the role of somatosensation in context-dependent learning and memorizing of pheromone locations remains unexplored. We addressed this problem by training female mice on a multimodal task to locate pheromones by sampling volatiles emanating from male urine through the orifices of varying dimensions or shapes that are sensed by their vibrissae. In this novel pheromone location assay, female mice’ preference toward male urine scent decayed over time when they were permitted to explore pheromones vs neutral stimuli, water. On training them for the associations involving olfactory and whisker systems, it was established that they were able to memorize the location of opposite sex pheromones, when tested 15 days later. This memory was not formed either when the somatosensory inputs through whisker pad were blocked or when the pheromonal cues were replaced with that of same sex. The association between olfactory and somatosensory systems was further confirmed by the enhanced expression of the activity-regulated cytoskeleton protein. Furthermore, the activation of main olfactory bulb circuitry by pheromone volatiles did not cause any modulation in learning and memorizing non-pheromonal volatiles. Our study thus provides the evidence for associations formed between different sensory modalities facilitating the long-term memory formation relevant to social and reproductive behaviors.

## 1 Introduction

In nature, when terrestrial rodents scour and encounter pheromones streaked on stones of different sizes and shapes, burrows and fallen leaves, how do they use different modalities to form a memory of the location of pheromones? Rodents are primarily macrosmatic mammals whose daily vital activities of foraging, finding mates, and avoiding predators are dependent on olfaction.^1^ However, when it comes to memorizing scent marks, is their sense of smell enough or are associations being formed between multiple senses, integrating information to retain the memory of the marks? In rodents, coordinated action of sniffing and whisking has been observed during the exploratory behaviors.^2,3^ However, it has not been tested if such multi-sensory actions facilitate the long-term memory formation of pheromones’ locations. We decided to undertake an approach for probing the role of different systems governing this by using a newly designed “multimodal pheromonal learning” behavioral paradigm. Our paradigm lets the female mice use sensory information processed by whisker pathway to associate a location presented with volatile and non-volatile pheromones over a location presented with water, a neutral stimulus. Although the female mouse is allowed to have a direct exposure to soiled bedding, it can sample the volatiles emanating from male urine through a plate guarded by orifices of circular shapes with specific diameters or of triangular shapes. This paradigm offers us to probe for the role of whiskers and microvibrissae present on the snout along with the olfactory subsystems in forming the memory of pheromone locations.

Classical knowledge reveals that pheromonal detection happens via vomeronasal organ (VNO) and main olfactory epithelium (MOE) which further project to accessory olfactory bulb (AOB) and main olfactory bulb (MOB), respectively. The output neurons project to bed nucleus of stria terminalis (BNST) and “vomeronasal amygdala” and further to hypothalamic regions, which control the lordosis behavior in females.^4–6^ Using genetic and lesion-based approaches, it has been found that AOB senses non-volatile pheromones present in male urine and soiled bedding through its VNO receptors (vomeronasal type 1, vomeronasal type 2, and formyl peptide receptors)^7^ while the trace amine-associated receptors present on MOE projecting to MOB output neurons detect air-borne, volatile pheromones.^8^ Another receptor type includes membrane-spanning 4-domains subfamily A (MS4A) receptors of “necklace” glomeruli subsystem found in caudal MOB, which are activated by certain pheromones such as 2,5-dimethylpyrazines. ^9^ Such parallel mechanisms occurring via different olfactory subsystems reflect the complexity of pheromonal information processing by activating combination of receptor subtypes in rodents.^10^

Male urine scent marking is an excellent example of intra- and inter-sex social communication^11^ leading to display of dominance by the giver^12,13^ or attractive/preferential behaviors by recipient females.^14^ In the context of mating, pheromones are important to be learnt and remembered by female mice. Earlier studies suggested that repeated vagino-cervical stimulation along with the pheromonal exposure is required for acquisition of long-term memory in females.^15^ Although, now we know that a single exposure is enough to form a memory,^14^ we do not yet understand the involvement of different sensory modalities in governing the memory formation/acquisition.

Our paradigm involves probing the effect of pheromone priming on the chemo- as well as whisker-mediated investigatory behavior and long-term memory formation in female mice. We observed that the preference toward a zone containing pheromones decayed over time when mice were freely exploring the arena during the initial 4 days. Furthermore, we trained female mice to learn the associations involving male pheromones, both volatiles and non-volatiles, and whisker-mediated sensory cues. These mice exhibited long-term memory when tested 15 days after the training, in the absence of any sensory cues. In order to investigate if somatosensation is important for locating such marks, we checked if the preference toward male mouse pheromones is negatively affected when the sensory input through whiskers and skin is blocked during the 15 days of training. Interestingly, we saw poor pheromonal long-term memory in these females suggesting the involvement of somatosensory system along with main and accessory olfactory systems. To investigate the differential activity-dependent neuronal activation across associative brain regions under sensory intact and deprived conditions, we examined the expression of activity-regulated cytoskeleton (Arc) protein. Significantly higher Arc protein expression was found in the somatosensory barrel cortical layers and hippocampi of mice whose sensory inputs were not blocked. This activation was found out to be highest in the case of whisker intact mice displaying multimodal memory on day 15th toward attractive opposite sex pheromones. Such an increased and specific activation of brain regions support our finding of the robust pheromonal memory formation. To probe if pheromone-dependent activation of main olfactory system can modulate other olfactory-driven behaviors, we carried out volatile discrimination learning and memory tasks. The pheromonal exposure induced Whitten effect, however, did not cause any differences in the MOB mediated discrimination learning pace for various non-pheromonal volatiles. Our data thus provide evidences for novel associations between different sensory modalities in facilitating long-term memory formation of pheromone locations.

## 2 Materials and Methods

### 2.1 Subjects

A total of 96 C57BL/6J female (10-14 weeks, Jackson Laboratories) and 10 C57BL/6J male mice (12-14 weeks, Jackson Laboratories) were utilized for the study. Three different groups of female mice were tested on “multimodal pheromonal learning” paradigm. Group 1 consisted of eight whisker intact female mice trained for associating Male soiled Bedding and Urine volatiles (MBU) with a particular diameter punched on the plate guarding the chamber 1, where the stimulus is kept. Group 2 had eight female mice, whose whiskers were trimmed during the training phase for the associations involving MBU. But with the same group, whiskers were kept intact during initial testing and memory testing phases. Group 3 consisted of eight whisker intact female mice trained for associating Female soiled Bedding and Urine (FBU) with a particular diameter. Group 4 consisted of eight whisker intact female mice trained for associating MBU and neutral stimuli with the distinct shape (either triangle or circle of perimeter/circumference of 31.4 mm) of the plate guarding the chamber. Four mice each from groups 1, 2, and 3 were utilized for measuring sniffing behavior toward pheromonal volatiles. For a given experiment, male urine was collected from either one male mouse or two mice housed together (C57BL6/J, aged 10-12 weeks), who never had a sexual experience. Female urine was collected from two female mice housed together (C57BL6/J, aged 10-12 weeks), who were also never sexually experienced. Soiled bedding was collected from the respective cages, just before the commencement of the experiment. For Arc immunohistochemical analyses, two to three animals from different experimental conditions were utilized to calculate the number of Arc positive cells in different brain regions.

For non-pheromonal odor discrimination training, 30 female mice, aged 8-14 weeks, were used. These were divided equally into two groups, one group (experimental, group 5) exposed to MBU every day during the discrimination training period while the other group (control, group 6) unexposed. Twelve-hour light/dark cycle was maintained and mice were grouped in individually ventilated cages in a temperature- and humidity-controlled animal facility. All animal care and procedures were in accordance with the Institutional Animal Ethics Committee (IAEC) at IISER Pune and the Committee for the Purpose of Control and Supervision of Experiments on Animals (CPCSEA), Government of India.

## 3 Multimodal Pheromonal Learning

### 3.1 Apparatus

**Figure 1:**
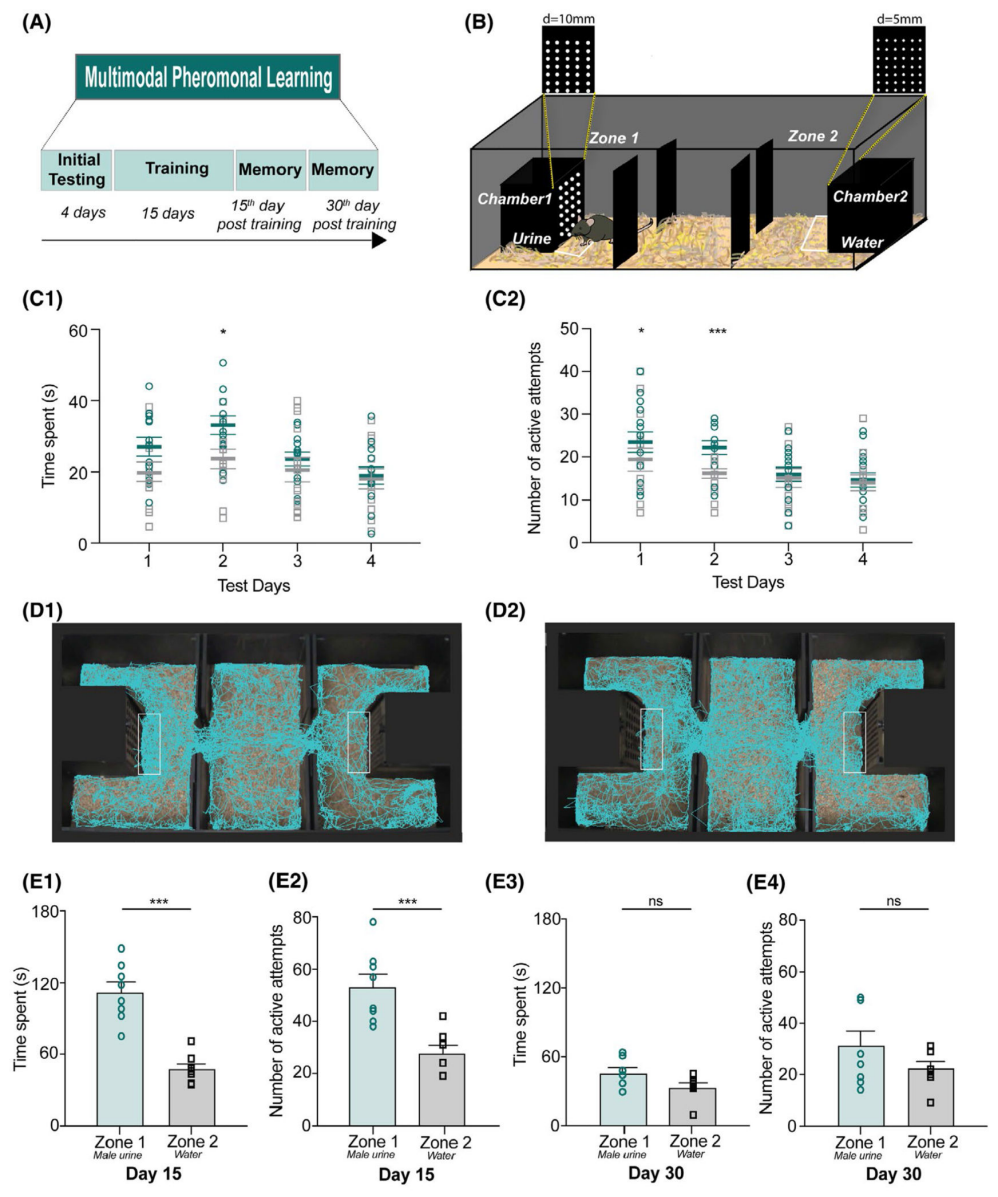
Multimodal associative learning causes long-term memory formation of pheromone locations in female mice. A, Experimental design of “multimodal pheromonal learning” paradigm. It involves an initial testing phase of 4 days, training phase of 15 days and the testing of memory at 15th and 30th day post-training. B, Diagrammatic representation of the set-up used for the assay. Female mouse is shown to be sensing the volatiles emanating through the specific diameter orifices from the stimulus present in OSP chamber. Perimeter demarcated by white line represents the zone where time spent by mouse is calculated using the EthoVision software by tracking the “nose point” of the animal. C1, Similar time spent near OSP chamber (teal colored symbols) vs NS chamber (gray colored symbols) on day 1, 3, and 4 of testing phase. Increased time spent on day 2 indicates emerging preference toward OSP, which, declines as the days of exploration progress. (*P* > .05, *F* = 4.34 repeated measures (RM) two-way ANOVA; Bonferroni’s multiple comparison test, *P* < .05 for day 2, *P* = .1 for day 1, *P* > .9 for day 3 and day 4; N = 13 mice). C2, Similar number of active attempts on plate guarding OSP chamber vs NS chamber on day 3 and 4 during initial testing phase. On day 1 and day 2, mice exhibited more number of active attempts manifesting initial preference toward OSP, which was reduced on day 3 and 4 (*P* > .05, *F* = 4.1 RM two-way ANOVA; Bonferroni’s multiple comparison test, *P* = .016 for day1, *P* = .0003 for day 2, *P* > .9 for day 3 and day 4; N = 15 mice). D1 and D2, Tracks recorded using EthoVision software by tracking the “nose point” of the animal. Visibly more time spent near zone 1 compared to zone 2 in case of memory day 15th (D1) while equal time spent for both the zones in case of memory day 30th (D2) is seen. The area demarcated is used for calculating time spent near the two chambers. E1, More time was spent near OSP chamber than NS chamber on day 15 post-training period (*P* = .0002, paired two-tailed student’s t test, N = 8 mice). Memory was tested in the absence of any stimuli (urine, soiled bedding, and water). E2, Number of active attempts on plate guarding OSP chamber was higher than on NS chamber on day 15 post-training period (*P* = .0004, paired two-tailed student’s t test, N = 8 mice). Memory was tested in the absence of any stimuli (urine, soiled bedding, and water). E3, Similar amount of time was spent near OSP chamber and NS chamber on day 30 post-training period (*P* = .109, Wilcoxon matched pairs signed rank test, N = 7 mice). Memory was tested in the absence of any stimuli (urine, soiled bedding, and water). E4, Similar number of active attempts were made on plate guarding OSP chamber and NS chamber on day 30 post-training period (*P* = .125, Wilcoxon matched pairs signed rank test, N = 8 mice). Memory was tested in the absence of any stimuli (urine, soiled bedding, and water)

It consisted of an arena (60 cm × 30 cm × 15 cm, length × width × height, coated with non-reflective black spray paint), which was divided into three equally spaced zones. Two 10 cm × 10 cm × 15 cm chambers guarded by removable plates were placed at the opposite ends of the length of arena. One removable plate guarding a particular chamber had equidistant 10 mm diameter orifices on it, keeping other three sides of each chamber devoid of any orifices. Chamber on the opposite side was guarded by a removable plate having equidistant 5 mm diameter orifices. This allows the animal to sample the volatiles emanating from the chamber through only one (front) side. One of each kind guards these chambers when the animal was sampling the volatiles during the initial testing and training phases of the experiment. The apparatus was custom-built using black acrylic sheets (6 mm thickness for the walls and 10 mm thickness for the base). Each chamber contained a 55 mm petri dish filled with 100 μL urine (attractive pheromonal stimulus in chamber 1) or water (neutral stimulus in chamber 2) (Figure 1B). Two different apparatuses were used for carrying out the multimodal pheromonal learning paradigm in the MBU and FBU groups. For shape-based multimodal pheromonal learning task, one of the removable plate guarding one chamber had triangle-shaped orifice while the other plate had circle shaped orifices of 10 mm diameter. The orifice dimensions were chosen such that the circumference of the circular orifices matches with the perimeter of the triangular orifice on the opposite sides.

### 3.2 Paradigm

The experimental design involves an initial testing phase of 4 days, training phase of 15 days and the testing of memory at 15th day and 30th day post-training (Figure 1A). The initial testing phase was to investigate if female mice exhibit innate preference toward a zone presented with attractive volatile and non-volatile pheromones from male mice (zone 1) over a zone containing neutral stimulus, water (zone 2). To remove any directional bias toward a particular zone, the apparatus was rotated by 180° every day during the initial testing and training phases. Mice were counterbalanced for the association with a specific diameter for circular shapes and the volatile cue pairing (size group, urine/5 mm vs water/10 mm and urine/10 mm vs water/5 mm, groups 1, 2, and 3) and for triangular or circular shapes and volatile cues (shape group, urine/triangular-shaped orifices vs water/circular-shaped orifices, and urine/circular-shaped orifices vs water/triangular-shaped orifices, groups 4) to remove any possible bias toward specific diameter or shapes. The chambers remained the same during this counterbalancing, although new plates were used. Training was done following the initial testing phase for a period of 15 days. Each day, the animal was restricted in both the zones for a period of 15 minutes each (alternating between the two zones after every 5 minutes). To check for the multimodal memory on 15th and 30th day, all volatile and non-volatile pheromonal stimuli (urine and the soiled bedding with non-volatile pheromonal traces) and neutral water stimuli were removed from the chambers of the apparatus while keeping the plates of specific diameter orifices undisturbed. The time spent in each of the zones, specifically in front of chamber 1 and chamber 2 was calculated using EthoVision software (Noldus Information Technology). The nose point feature (used to track animal) on EthoVision is used to visualize the tracks taken by an animal (Figure 1D1,D2) and to calculate the time spent. Number of active attempts on the plates guarding chamber 1 and 2 were calculated manually by considering single nose poke into the plate as one attempt. Multimodal memory was also tested in a new apparatus and plates, which were identical in dimensions to the apparatus used for the training phase. This was done to ensure that the apparatus and plates are devoid of any residual pheromones.

In case of testing the involvement of somatosensation in pheromone location learning and memory, an anesthetic gel (Lignocaine Hydrochloride gel- Lox-2% jelly, Neon laboratories) was applied on the snout of the group 2 mice during the training phase of 15 days. Whisker trimming was done at a frequency of 5 days from the start of the training phase. Whiskers were allowed to grow and were never trimmed again after the training period. Mice had re-growing whiskers when the day 15 memory was tested, thereby allowing us to specifically check for the role of somatosensation in mediating the association of pheromonal cue with size. Additionally, a separate group of mice whose whiskers were only trimmed were used to carry out multimodal pheromonal learning paradigm. The anesthetic gel was not applied during the training period to assure that there is no confounding effect of the gel on olfactory sensation.

## 4 Go/No-Go Odor Discrimination

### 4.1 Odors

Odors used were 1,4-Cineole (CI), Eugenol (EU), Amyl acetate (AA), Ethyl butyrate (EB), Benzaldehyde (BZ), Nonanol (NN), Hexanal (HX), and 2-Pentanone (PN). The odors were diluted to 1% in mineral oil and further diluted 1:20 by airflow. All odors were bought from Sigma-Aldrich and mineral oil was bought from Oswal Pharmaceuticals, Pune, Maharashtra, India.

### 4.2 Apparatus

All experiments were done using two custom-made eight channel olfactometers controlled by software written in Igor Pro (Wavemetrics, OR). Briefly, mouse was put into an operant chamber, which had a combined odor sampling and reward delivery port on one end of the chamber. This ensured tight association of reward with the odor presented in a trial. Inside the odor port, a lick tube was placed on which the animal needs to lick to get the reward. Insertion of animal’s head in the port resulted in breaking of an infra-red (IR) beam guarding odor/reward port, which initiated the trial. The odor and final (diversion) valves controlled the flow of odor in a time-dependent manner, allowing opening of final valve 500 ms after odor valve opening. Each rewarded (S+) and non-rewarded (S-) odor was presented through either of the two valves. A total of four odor valves for two odors were used. The apparatus and the task design followed is same as done in previously published studies (Figure 5C1-C3).^16–19^

### 4.3 Task habituation phase

First week post-water restriction involved training with the standard operant conditioning procedures. Initially, mice were rewarded with water upon insertion of head into the sampling port and breaking the IR beam. The difficulty levels increased gradually, wherein the animal needed to lick on the tube to get the reward. At the end of this phase, odor valve was introduced and the air passing through the mineral oil (solvent used for odorants) bottle was used as the stimulus.

### 4.4 Discrimination training phase

As the animal initiated the trial by breaking the beam, one of the odor valves and a diversion valve (DV) open for certain duration. The DV helps to divert the odorized air to the exhaust for 500 ms and thereafter to the odor port for the pre-decided stimulus duration (2 s for the odor discrimination training). For a rewarded (S+) odor to be correctly registered, animal needs to lick any three of four bins of 500 ms to get the reward. For an unrewarded odor (S-) to be correct, animal is allowed to lick at most two of four bins. No punishment is given for a wrong response toward S- odor. There is a fixed inter-trial interval (ITI) of 5 s during which the animal cannot initiate the next trial. Usually an optimally motivated animal took more than 10 s as the ITI. Odors were presented in a pseudo-randomized manner (not more than two successive presentations of same odor and S+/S- odors were distributed equally in a block of 20 trials).

### 4.5 Resistance to memory extinction task

Once the animals reached a criterion performance of >80% for an odor pair for which long-term memory needs to be checked, we performed resistance to memory extinction task. This was done to stabilize the memory of an odor pair and is based on the “Partial reinforcement theory”.^20^ The task comprised of 100 trials with 50 trials for S+ and S- each. The S+ trials are reinforced pseudo-randomly only for half of the trials as compared to a normal reinforcement learning where all S+ trials are rewarded. Such a partial reinforcement learning increases the attention of the animal as the factor of anticipating a reward is no longer true for some trials and that the trial outcomes cannot be expected by the animal. This increases the association strength and helps preventing memory extinction.

### 4.6 Memory task

The olfactory memory was checked for AA vs EB, 30 days after carrying the resistance to memory extinction task. The memory task comprised of 200 trials, first 60 trials of background odor pair and subsequent 140 trials with inter-leaved memory trials [4 trials (two S+ and two S- trials) in every block of 20 trials of background odor pair]. The memory task was performed only after the accuracy of background odor pair reached >80% for all animals. For checking AA vs EB memory, BZ vs NN was used as a background odor pair. Memory trials were not rewarded. Memory is calculated as an average accuracy percent of 28 trials (14 S+ and 14 S- trials) tested over a total of 140 trials.

### 4.7 Sniffing behavior toward pheromones

To investigate the sniffing strategies of female mice toward male urine and female urine volatiles under whisker trimmed vs intact conditions, a head-restraining method was used to precisely deliver the volatiles on the snout of the mice.^21^ These were subsets of mice utilized for multi-modal pheromonal learning assay and their sniffing strategies were tested 2 months after their 30th day memory was investigated. They were implanted with a head-post (custom-built head fixation set-up) on their head. The skin overlaying the skull was removed and head post was fixed using dental cement (Tetric N-Ceram, Ivoclar Vivadent). Surrounding exposed skull was covered using the dental acrylic cement (DPI RR Cold cure, acrylic repair material). This surgery was performed under anesthesia maintained by intraperitoneal injection of Ketamine (Celtamin, Celtiss therapeuticals) 60 mg/kg and Xylazine (Vea Impex) 10 mg/kg. The eyes were hydrated with 1% (w/v) carboxymethyl-cellulose (Refresh liquigel, Allergen India) to prevent dryness. Recovery time of 2 days was given and the weights were measured every day. Upon recovery, they were placed in a plastic tube and head-post was screwed onto a metallic device fitted on a platform. Urine volatiles and air presentations were done in a pseudorandom fashion through a nozzle. One hundred microliter of urine was used for the same. Their sniffing behavior toward the volatiles was recorded using airflow pressure sensor (AWM2300V, Honeywell) placed near one of the nostrils.^21^ Each presentation was carried out for a period of 2 s repeated over 10 such presentations of the same stimulus (total 20 presentations), accounting for a total of 20 s time of exposure to pheromonal volatiles on a single day.

### 4.8 Immunohistochemistry

Mice were sacrificed using Thiopentone (Thiosol sodium, Neon laboratories, 50 mg/kg) and perfused using a fixative. Transcardial perfusion was done using 50 mL of 1X Phosphate-buffered saline (PBS) followed by 4% (w/v) Paraformaldehyde (PFA). Brain of the animal was dissected and incubated in 4% PFA overnight at 4°C. Next day, tissue was washed twice with 1X PBS to remove extra fixative. In order to carry out cryotome sectioning, brain was cryopreserved using 20% sucrose (w/v) for a day and put on a rotator (Tarsons rotaspin). It was then transferred to 30% sucrose (w/v) at 4°C overnight. Brain tissue was embedded in optimal cutting temperature (OCT) medium (Leica, 14020108926) and sectioned in a cryotome. Fifty micrometer coronal sections were cut. Sections were washed in 1X Tris-buffered saline (TBS) twice for 10 minutes each in a six-well plate. Blocking solution (5% bovine serum albumin (BSA) and 1% Triton-X in TBS) was added to the wells and plate was kept undisturbed for 1.5 hours. Sections were then incubated in primary antibody (Rabbit anti-Arc, Arc-156003, Synaptic Systems) diluted 1:1250, in blocking solution (1% BSA, 0.1% Triton-X in TBS) for 14 hours in 4°C. TBS washes (thrice, 15 minutes each) were given to remove the excess of antibody. Incubation with secondary antibody (Anti-Rabbit Alexa Fluor 594, Jackson’s immunoresearch) diluted 1:500 in 1% BSA in TBS and kept for 2 hours at room temperature. Sections were then washed with TBS (thrice, 15 minutes each) and labeled with DAPI (4’,6-diamidino-2-phenylindole) (Sigma, 1:500). They were mounted on glass slides using Vectashield anti-fade mounting medium (Vector labs, H-1000).

### 4.9 Confocal Imaging and cell count quantification

For quantification of Arc labeled nuclei across the coronal brain sections, imaging using SP8 confocal microscope (Leica) was done (a total of 4-8 50 μm sections were utilized for quantification for each brain region, per animal). Quantification of the Arc positive cells was done using Imarisx64 software (Bitplane, Oxford Instruments). Upon uploading the z-stack (captured at 1 μm optical section) in the software, only the channel corresponding to Arc signal was selected. The 3D stack was then passed through a fixed value Gaussian filter to reduce the background noise. A constant XY diameter was applied and the threshold was automatically set by the software. The cells were counted through the thickness of the stack and an output cell density number was generated. For each brain region, total cells were calculated by summing the cell counts of each of the z-stacks per animal. The total volume was calculated by multiplying the imaged area with thickness of the stack acquired (∑ of sampled area in mm^2^ × stack thickness in mm). The cell count, for each region, was finally represented as the total number of Arc positive cells in 1 mm^3^. The volume in which Arc positive cells were visualized are scanned and considered for quantification throughout the manuscript.

### 4.10 Statistical analyses

All data are represented as mean ± SEM (standard error of mean) and analyzed using Graphpad Prism 8.0 (Graphpad Software Inc, USA). Normality of the data was checked using Shapiro-Wilk test (for all data with N = 3 and above). For data that followed normal and lognormal distribution, unpaired student’s t test, paired student’s t test, one-way analysis of variance (ANOVA) and two-way ANOVA with *post hoc* Bonferroni’s multiple comparison testing were carried out wherever required. All t tests were two tailed. For data that did not follow normal distribution, Mann-Whitney and Wilcoxon matched pairs signed rank tests were carried out wherever required. *represent *P* < .05, **P < .01, ***P < .001, and ****P < .0001.

## 5 Results

### 5.1 Multimodal associative learning causes long-term memory formation of pheromone locations

We designed a “multimodal pheromonal learning” paradigm to test the preference and acquisition of memory of female mouse toward the urine of opposite sex (Figure 1A). It involved a set-up consisting of zone 1 and zone 2. A 55 mm petri dish containing male urine was kept in the chamber 1 [used henceforth as opposite sex pheromones (OSP) chamber] of zone 1. Neutral stimulus (NS), water was kept in a petri dish in chamber 2 [used henceforth as NS chamber] present in zone 2. A mouse can sample the volatile stimuli through specific diameter orifices made on one side of the chamber as depicted in Figure 1B. To avoid any bias toward specific chamber, diameters were counterbalanced across mice for sensing male urine and water. Separate plates of a particular diameter were used in these conditions.

To investigate mice’ (group 1) innate preference toward any zones, they were allowed to explore both zones for a total of 10 minutes every day during the initial 4 testing days. To remove any possible directional bias shown by the mice toward a particular zone, the apparatus was rotated by 180° every day during the initial testing and training days (See Materials and Methods). The preference was quantified based on the time animal spent in front of OSP chamber sensing the non-volatile pheromones from male soiled bedding (region demarcated by a white line in Figure 1B) and the volatiles emanating from the OSP chamber. Mice made repetitive snout pokes into the orifices of the plate guarding the chambers to sense the stimuli. Such an experimental design facilitated the multimodal learning of pheromone locations with the stimuli. Thus, another parameter to measure the preference was the number of active attempts or snout pokes into the orifices of specific diameter to sense the volatiles. Decayed preference was observed over the course of 4 consecutive initial testing days. On day 2 of the initial testing phase, we observed an increase in the time spent toward OSP chamber indicating the emerging preference toward male urinary pheromones, which was reduced during the remaining 2 days (Figure 1C1: *P* > .05, *F* = 4.34 repeated measures (RM) two-way ANOVA). (Figure 1C1: RM two-way ANOVA, Bonferroni’s multiple comparison test, *P* < .05 for day 2 and *P* > .05 for all other days). Similarly, on initial days, that is, day 1 and day 2, female mice made higher number of active attempts to sample the urinary volatiles. The number of attempts, however, became similar to the exploration behavior on water side as the days progressed (Figure 1C2: *P* > .05, *F* = 4.1, RM two-way ANOVA; Bonferroni’s multiple comparison test, *P* < .02 for day 1 and day 2, *P* > .9 for day 3 and day 4). To test if this overall decline is due to variable responses shown toward both stimuli, we quantified and compared their attraction specifically toward the pheromones. Indeed, female mice spent lesser time (Figure S1A1: *P* < .01, *F* = 6.3, RM one-way ANOVA; Bonferroni’s multiple comparison test, *P* < .05 for day 2 vs day 3 and day 2 vs day 4) and made fewer active attempts particularly toward OSP chamber as the days progressed during the initial testing phase (Figure S1A2: *P* < .0001, *F* = 16.3, RM One-way ANOVA; Bonferroni’s multiple comparison test, *P* < .01 for day 1 vs day 3, 4 and day 2 vs day 3, 4).

In order to investigate if the association of non-volatiles and volatiles through olfactory and whisker systems can be strengthened over time, we further trained mice for a period of 15 days after their initial testing period was over. During this time, a mouse is allowed to enter only one of the zones and the door is then closed for 5 minutes ensuring the intended associative learning of pheromone locations with the volatiles and the specific orifice size. This is then alternated for both sides leading to a total of 15 minutes being spent on each side. Indeed, this restriction to one particular zone allowed mice enough time to explore the size, sense the volatiles, and the non-volatiles from soiled bedding. To investigate if such a multimodal association can be retained, we checked for pheromonal memory at day 15 and day 30 post-training phase. Exposure to OSP was not allowed during the time period after the training. We observed an increased preference toward OSP chamber on day 15, as measured by the two parameters: time spent and number of active attempts (Figure 1D1,D2). This confirms that mice had intact memory at day 15 (Figure 1E1,E2, *P* < .001, paired two-tailed student’s t test) after the multimodal training was carried out. To assure that there are no confounding effects arising because of residual pheromones in the apparatus, we tested the memory of a separate group of mice in a new but identical apparatus (Figure S2A). The plates and the apparatus dimensions were identical to those used in the training phase. On memory day 15th, mice showed a preference toward the OSP chamber compared to the NS (Figure S2B, *P* = .01 for time spent and S2C, *P* = .003 for number of active attempts, paired two-tailed student’s t test).

We did not observe any preference when the 30th day memory was analyzed (Figure 1E3: *P* > .05 for time spent and Figure 1E4: *P* > .1 for number of active attempts, Wilcoxon matched pairs signed rank test) after the training. As mice were not exposed to the stimuli for an extended period of 30 days, it possibly led to extinction of preferential memory for the attractive stimulus.

### 5.2 Pheromone location memory formation is facilitated by the association formed between olfactory and whisker systems

**Figure 2:**
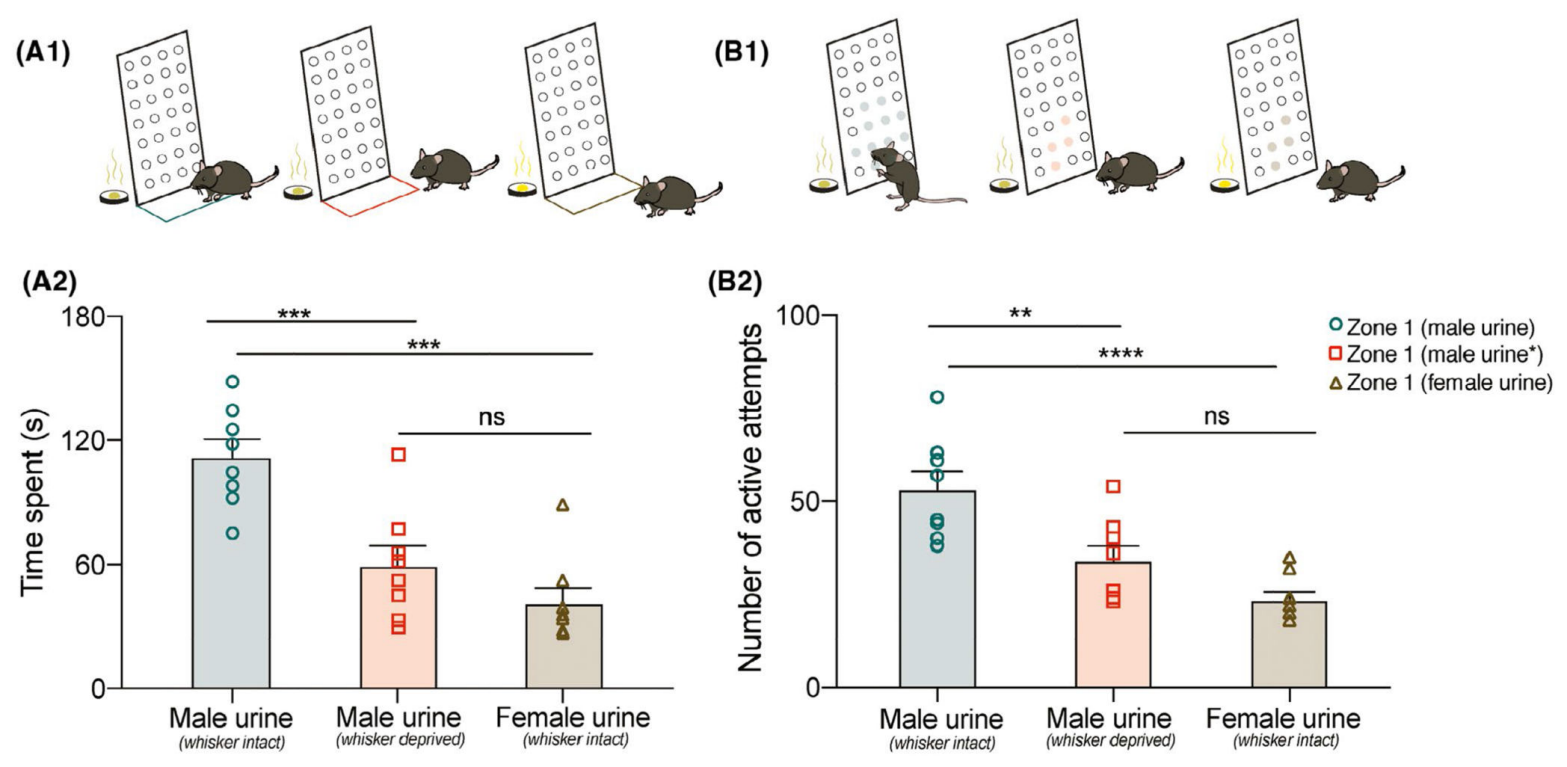
Long-term memory of pheromone location is facilitated by the association formed between olfactory and whisker systems. A1, Schematic representing a female mouse sampling in front of the plate guarding OSP chamber in the perimeter demarcated by a colored line. Nose point of the mouse snout is tracked for measuring parameter “time spent” by EthoVision software (Teal: whisker intact female mouse trained toward MBU, Red: whisker deprived mouse toward MBU and Brown: whisker intact mouse toward FBU). Memory was tested in the absence of any stimuli (urine, soiled bedding and water). A2, Reduced memory was observed for whisker deprived female mice trained with male urine and whisker intact female mice trained with female urine compared to whisker intact female mice trained with male urine on 15th day memory testing (*P* < .0001, *F* = 18.69, ordinary one-way ANOVA, Bonferroni’s multiple comparison test; Male urine vs Male urine*: *P* = .0005, Male urine vs Female Urine: *P* < .0001, Male urine* vs Female urine: *P* > .1, N = 8 mice for all groups) (*whisker deprived). B1, Schematic representing a female mouse making active attempts by poking its snout multiple times into the orifices of a particular diameter on plate guarding OSP chamber. Color of the orifices’ circumference depict the experimental condition; teal: whisker intact group trained towards male urine; red: whisker deprived group toward male urine and brown: whisker intact group toward female urine. Memory was tested in the absence of any stimuli (urine, soiled bedding and water). B2, Reduced number of active attempts were observed for whisker deprived female mice trained with male urine and whisker intact female mice trained with female urine compared to whisker intact female mice trained with male urine on 15th day memory testing (*P* < .0001, *F* = 15.27, ordinary one-way ANOVA, Bonferroni’s multiple comparison test; Male urine vs Male urine*: *P* < .01, Male urine vs Female Urine: *P* < .0001, Male urine* vs Female Urine: *P* > .1, N = 8 mice for all groups) (*whisker deprived)

To systematically investigate if whisker system is being utilized as a result of repeated snout insertions into the plate with orifices of specific diameter during the training phase, we carried out whisker sensory deprivation in another set of mice (group 2, see Materials and Methods). In this experiment, whiskers were trimmed and an anesthetic gel was applied on the snout of the mouse during the training phase of 15 days so as to prevent the association of volatiles emanating from the stimulus kept inside the chambers guarded with plates of specific diameters. Topical, non-invasive application of anesthetic gel was done each day, 15 minutes before putting the mouse in the set-up for the training period. Toward the end of this recovery period, we observed normal mobility with all animals tested. The mice were then put in the multimodal pheromonal learning set-up (Figure 2A1,B1). Whiskers were not trimmed after the training period and all mice had re-growing whiskers on the day of memory investigation, day 15 post-training. The memory was significantly reduced in the whisker deprived group compared to whisker intact group indicating the involvement of whisker system in facilitating the multimodal learning and thereby the memory formation for pheromone locations (Figure 2A2: *P* < .0001, *F* = 18.69 ordinary one-way ANOVA; Bonferroni’s multiple comparison test, *P* = .0005 for time spent and Figure 2B2: *P* < .0001, *F* = 15.27 ordinary one-way ANOVA; Bonferroni’s multiple comparison test, *P* = .006 for number of active attempts). Indeed, we did not observe difference in the time spent in front of the OSP chamber vs NS chamber (Figure S3A1: *P* > .1, Paired two-tailed student’s t test) and the number of active attempts into the orifices on both the chambers (Figure S3A2: *P* > .1, Paired two-tailed student’s t test) suggesting that preference for attractive, opposite sex pheromonal stimuli was impaired for this group. We applied anesthetic gel on the snout of whisker trimmed mice to block the inputs from the micro-vibrissae and non-mystacial mechanoreceptors when the mouse makes active attempts onto the plates guarding the chamber during the training period. Lidocaine has been shown to cause reversible and transitory blocking of whisker induced activation when applied to the base of whiskers in rat.^22^ Thus, we had used this strategy to accomplish non-invasively, the complete blocking of somatosensation during behavioral training. However, to test if the anesthetic gel applied on the snout can interfere with the olfactory sensing abilities of mice which could impede acquisition of multimodal memory, we also carried out only one treatment of sensory deprivation, that is, whisker trimming only, the same way as done in group 2. We found out that whisker trimming alone also did not allow the mice to associate the size of the orifice with the attractive pheromone present inside the chamber. Mice spent equal times on both sides (Figure S3B1, *P* > .9, Wilcoxon test) and made similar number of attempts (Figure S3B2, *P* = .71, paired two-tailed student’s t test). This corroborates to the importance of tactile cues in generating sexual-physiological changes. For instance, social touch along with the pheromones can lead to accelerated puberty in female mice suggesting the relevance of tactile cues.^23^

Acquisition of memory of OSP location under whisker intact but not deprived conditions called for further interrogation. To study if the preference shown by female mice was specific to the opposite sex pheromones, we carried out multimodal training paradigm with “non-attractive,” same-sex pheromones using another set of female mice (group 3, see Materials and Methods). On testing the memory, we observed significantly lower response compared to the preference shown by group 1 mice (Figure 2A2,B2: *P* < .0001 for both parameters, ordinary one-way ANOVA, Bonferroni’s multiple comparison test). Furthermore, there is a possibility that mice might adopt varying sampling strategies for the same stimuli under whisker intact and deprived conditions, which can modulate their response. We investigated sampling behavior using subsets of female mice utilized for multimodal pheromonal learning assay. We analyzed the sniffing frequencies shown by whisker intact and deprived mice toward the volatile pheromonal cues during brief pheromonal exposure epochs (Figure S4A). These mice were head-restrained and urine volatiles were delivered using a custom-built olfactometer. Their sniffing strategies did not vary under different conditions for the time duration we tested for (Figure S4B: *P* > .1 across all groups on 4 consecutive days, ordinary one-way ANOVA). This confirms the fact that the reduced memory observed in group 2 and 3 is not due to sampling differences, but because of the robustness and specificity of associations formed between different modalities.

### 5.3 Sensory deprivation caused reduced neural activation patterns in olfactory and whisker systems

**Figure 3:**
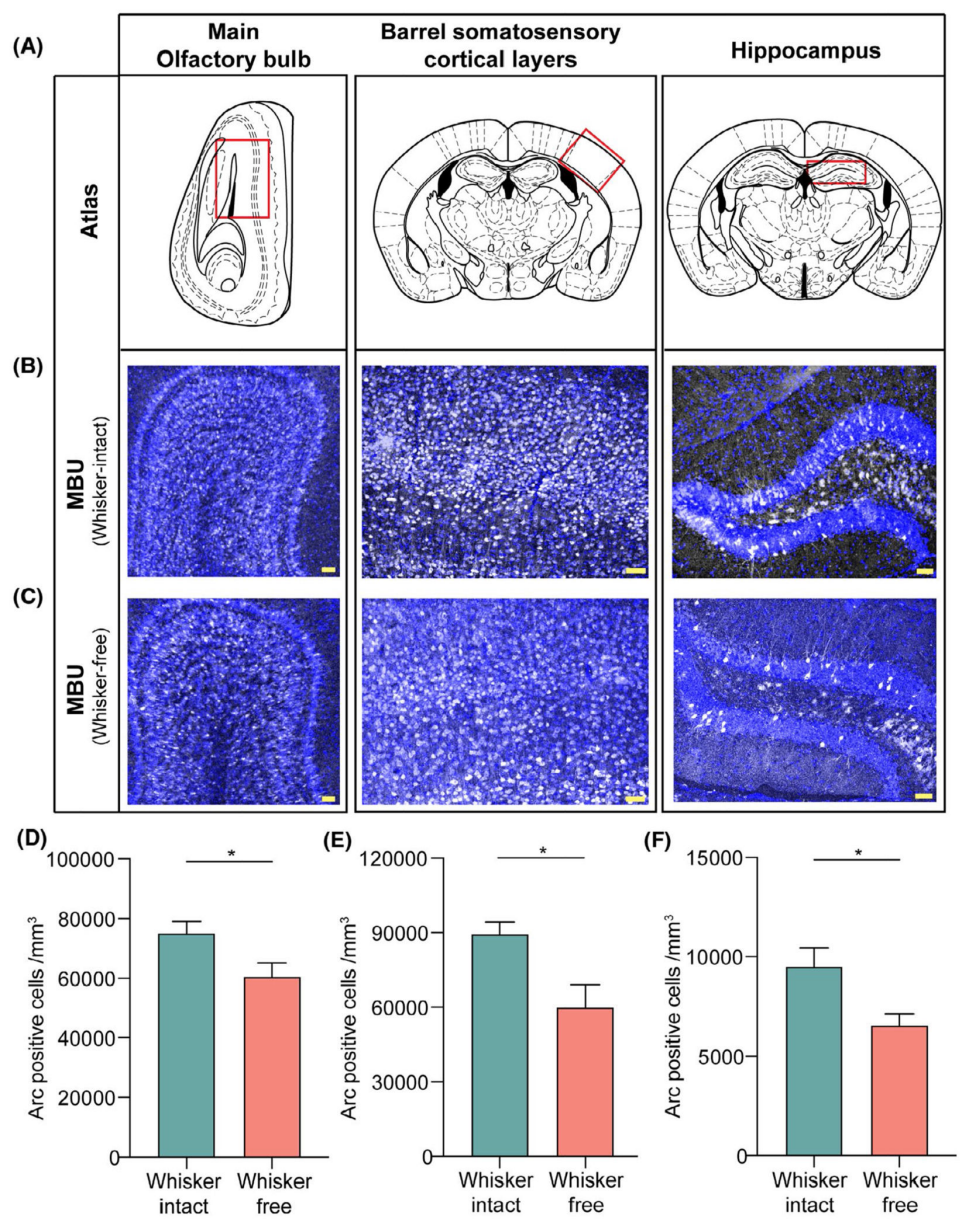
Arc immunoreactivity is lower in the olfactory bulb, barrel cortical layers and hippocampus of whisker free mice on memory day 15th. A, The panel shows atlas maps (adapted from The Mouse Brain in Stereotaxic Coordinates, George Paxinos) of the regions of interest (as mentioned on top) with red boxes demarcating the areas used for quantification of Arc positive cells. B, Immunofluorescence images of Arc immunoreactivity in the MOB, SSC and Hippocampus of whisker intact mouse on MD 15. Blue: DAPI, Gray: Arc, Scale Bar: 50 μm. C, Immunofluorescence images of Arc immunoreactivity in the MOB, SSC and Hippocampus of whisker free mouse on MD 15. Blue: DAPI, Gray: Arc, Scale Bar: 50 μm. D, Significantly lower Arc positive cells in the MOB of whisker free mice as compared to whisker intact mice (*P* = .03, unpaired two-tailed t test, N = 6 mice for each group). E, A substantial decrease in Arc positive cells in the SSC of whisker free mice as compared to whisker intact mice (*P* = .019, unpaired two-tailed t test, N = 6 mice for each group). F, Significantly lower Arc positive cells in the DG of whisker free mice as compared to whisker intact mice (*P* = .02, unpaired two-tailed t test, N = 6 mice for each group)

A recent study indicates that an initial sexual experience can lead to gain in the population of excitatory Scnn1a expressing neurons in layer 4 of genital cortex in somatosensory areas in pubertal female mice.^24^ As a starting point toward depicting the neural basis of learning and memory of pheromonal locations in our paradigm, we explored the activation pattern of Arc protein across days of training and testing memory. We analyzed the neural activation patterns in the olfactory bulb, somatosensory areas, and hippocampus of sensory intact and deprived animals immediately after the completion of multimodal learning and memory tasks. We found Arc positive cells present in the MOB, somatosensory cortex (SSC), and the dentate gyrus (DG) of hippocampal region of the MBU exposed whisker intact mice (Figure 3A displaying regions utilized for quantification). Such a specific activation profile of Arc indicates the involvement of olfactory and somatosensory neural circuits in pheromonal location learning and memory formation. First and foremost, we investigated if any alterations are occurring in the case when multimodal memory is either present or absent (Figure 3B,C). We found out that the absence of memory under whisker deprived conditions correlates with the decreased number of Arc positive cells in MOB, somatosensory layers, and hippocampus (Figure 3D-F; 3D: *P* = .03, 3E: *P* = .019, 3F: *P* = .02, unpaired t test, two tailed), when checked on memory day 15th (MD 15). Such a differential activation in intact vs free groups (free group: include whisker deprived and trimmed mice) correlates with the impaired acquisition and retrieval of pheromonal memory in the whisker free group.

Even at the time of completion of the training period of 15 days in a multimodal pheromonal learning task, whisker intact group displayed enhanced Arc expression as compared to whisker deprived group on the last day of training, day 15th (TrD 15) (Figure S5; S5D: *P* = .02 for SSC and S5E: *P* = .031 for DG, unpaired two-tailed t test). Both groups received similar olfactory inputs during the training days as only whisker inputs were blocked in the deprived group. This was reflected in the similar Arc activity in MOB of these two groups (Figure S5C, *P* = .5, unpaired two-tailed t test). As the whisker intact mice displayed the memorized preference on MD 15 due to the association formed between two systems, we further compared the Arc activation patterns evoked by learning and memory formation. We observed enhanced activations in whisker intact mice immediately after the animals showed the preference memory (Figure S5; TrD15 vs MD15 in whisker intact mice: S5C, *P* = .003 for MOB and S5D, *P* = .02 for SSC, unpaired two-tailed t test), whereas such activations were absent in whisker deprived mice (Figure S5; TrD15 vs MD15 in whisker deprived mice S5C, D, *P* > .1, unpaired two-tailed t test). The role of hippocampus in encoding memory retrieval, however, remains unscathed in both groups as both whisker intact (Figure S5, TrD15 vs MD15 in whisker intact group, S5E, #, *P* = .07, unpaired two-tailed t test) and deprived showed a slight increase in Arc positive cells in DG on MD 15 (Figure S5, TrD15 vs MD15 in whisker deprived group, S5E, *P* = .01, unpaired two-tailed t test). However, delineating the neural circuit activations happening in hippocampus during the exploratory behavior in our experimental apparatus vs memory retrieval under sensory intact and deprived conditions need to be investigated further.

In order to determine the specificity of Arc activation patterns due to multimodal learning and preference memory towards MBU, we compared the trained animals with naïve singly caged, age-matching female mice. The naïve mice were singly housed for the time corresponding to the whole duration of the multimodal pheromone learning and memory behavioral task. We found very low number of Arc expressing cells in MOB, SSC, and the DG of hippocampal region of naïve mice (Figure S6, S6C and D *P* = .002 for MOB and SSC, S6E, *P* = .03 for DG, Unpaired two-tailed t test). The Arc expression was even heightened for the attractive stimulus (MBU) compared to FBU trained mice, except in the hippocampal region (Figure S7, MBU vs FBU, S7C, *P* = .013 for MOB, S7D, *P* = .011 for SSC, S7E, *P* = .23 for DG, Unpaired two-tailed t test, see Discussion).

**Figure 4:**
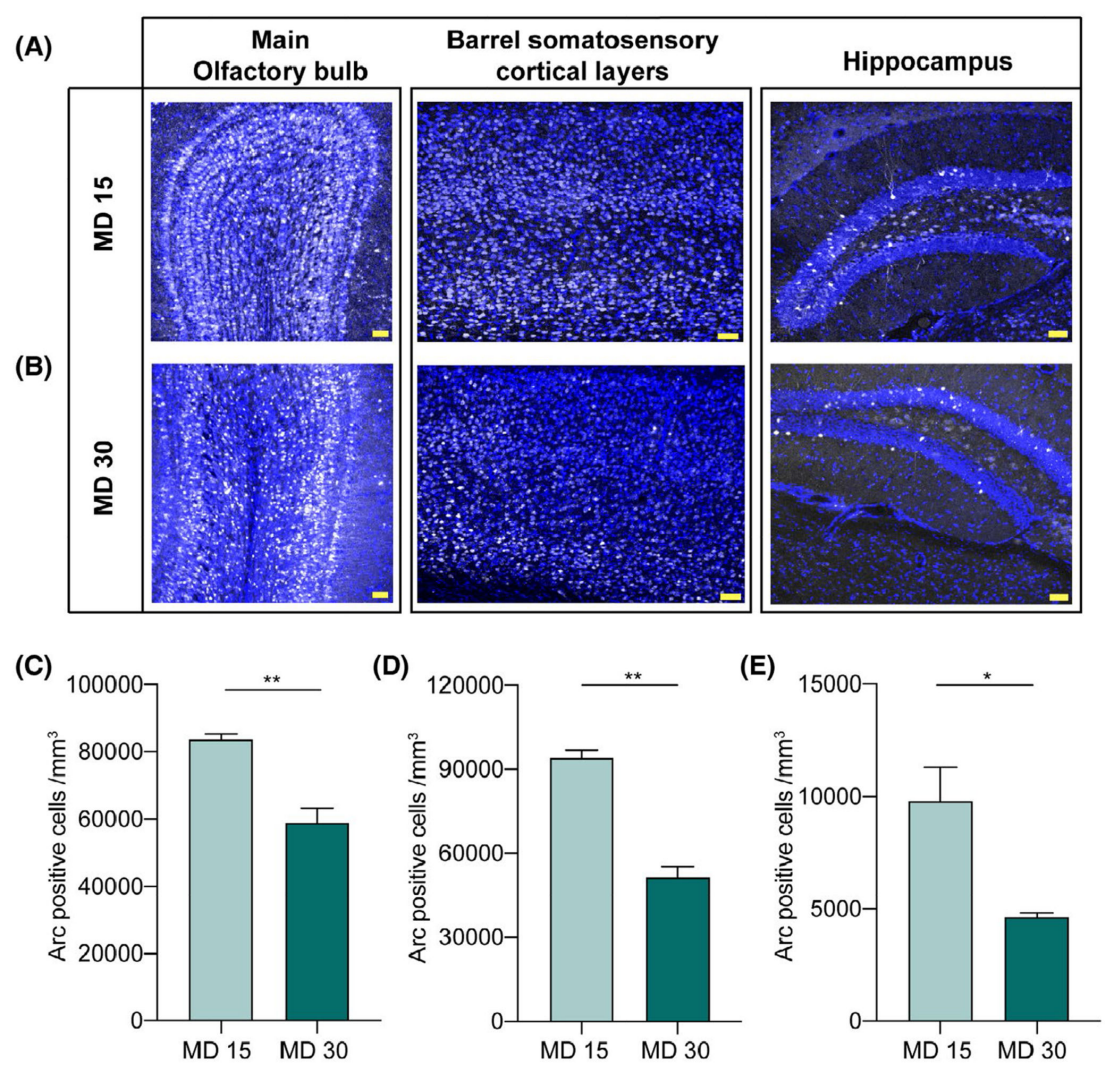
Decrease in Arc expression correlates with decay in memory from 15th day to 30th day. A, Immunofluorescence images of MOB, DG, and SSC barrel cortical layers on MD 15 (Blue: DAPI, Gray Arc, Scale bar: 50 μm). B, Immunofluorescence images of MOB, DG and SSC barrel cortical layers on MD 30 (Blue: DAPI, Gray: Arc, Scale bar: 50 μm). C, Significantly decreased number of Arc positive cells in MOB on MD 30 when female mice do not exhibit intact multimodal memory (*P* = .006, unpaired two-tailed t test, N = 3 mice). D, SSC immunoreactivity of Arc is decreased on MD 30 as compared to MD 15 (*P* = .0009, unpaired two-tailed t test, N = 3 mice). E, Hippocampal expression of Arc is decreased on MD 30 as compared to MD 15 (*P* = .025, unpaired two-tailed t test, N = 3 mice)

As the pheromone location memory was absent when tested on 30th day (Figure 1E3,E4), we further analyzed the Arc activation patterns after animals showed lack of memory (Figure 4A,B). Activated neuronal ensembles, as visualized by Arc, were not sustained in all the three regions on MD 30 when compared to MD 15 (Figure 4C-E, *P* = .006 for MOB, *P* = .0009 for SSC and *P* = .025 for DG). The absence of pheromonal exposure over a prolonged gap of 30 days after the training period, could have resulted in plasticity-dependent changes in MOB, thereby, forgetting the memory of pheromones on MD 30. Indeed, it has been shown previously that female mice cannot remember pheromonal locations for long term in the absence of repeated exposures.^25^ Expression of Arc in somatosensory cortical layers upon whisker stimulation and exploration conveys its role in faithfully revealing the specific ensembles activated. Hippocampus receives information multiple sensory modalities, including olfactory and somatosensory regions. We observed a decrease in Arc positive cells in the barrel cortical layers and DG of hippocampus on MD 30, which is concomitant with the reduced pheromone location memory.

### 5.4 Activation of main olfactory bulb circuits by pheromone volatiles does not modulate discrimination abilities toward non-pheromonal volatiles

**Figure 5:**
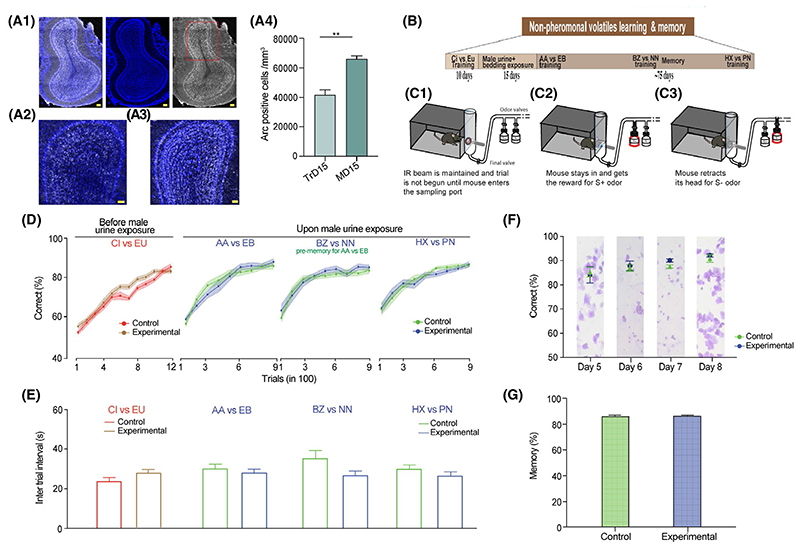
MOB circuitry activation by urinary volatiles does not modulate non-pheromonal volatile discrimination learning and memory. A1, Tilescan immunofluorescence image of Arc immunoreactivity in the MOB of whisker intact mouse exposed to MBU. Blue: DAPI, Gray: Arc, Scale Bar: 100 μm. A2, Image depicting Arc positive cells (gray) in the MOB (magnified granule cell layer) on training day 15th, Scale bar: 50 μm. A3, Image depicting an increased number Arc positive cells (gray) in the MOB (magnified granule cell layer) on memory day 15th, Scale bar: 50 μm. A4, An increase in the proportion of Arc immunoreactive cells in the MOB of whisker intact group from TrD 15 to MD15 (*P* = .003, unpaired t test, two-tailed, N = 3 mice for each group). B, Experimental design for the go/no-go associative olfactory learning training for MBU exposure and no-exposure female mice groups to investigate their olfactory discrimination learning and memory. C1-C3, Schematic of go/no-go odor discrimination paradigm indicating the sequence of events in a rewarded (C2) vs unrewarded trial (C3) for a trained mouse. D, Learning curves depicting accuracy of discrimination across different odor pairs before MBU and after MBU exposure, shown as percent correct choices of 100 trials. Mice from both the groups were trained for CI vs EU to ensure similar learning efficiencies before Whitten effect was induced (ordinary two-way ANOVA, Bonferroni’s multiple comparison test, *P* > .05 for all data points in learning curve). No difference in the pace of learning across different odor pairs was observed after Whitten effect was induced (AA vs EB: *P* > .1, *F* = 0.12; BZ vs NN: *P* > .1, *F* = 1.43; HX vs PN: *P* > .1, *F* = 0.19; ordinary two-way ANOVA, Bonferroni’s multiple comparison test, *P* > .05 across all data points for all learning curves, N = 11-15 mice for both groups across odor pairs). E, Similar inter-trial interval, a readout of motivation levels shown by animals of both groups across different odor pairs, while performing the behavioral task. CI vs EU: *P* = .2, Mann-Whitney test; AA vs EB: *P* = .42, BZ vs NN: *P* = .06, HX vs PN: *P* = .26, unpaired two-tailed student’s t test, N = 11-15 mice for both groups across odor pairs. F, Average discrimination accuracy values during four consecutive days of AA vs EB training done after the induction of Whitten effect. On each of these days, the accuracy was similar between experimental and control group. Vaginal cytology in background for each day on the plot depicts induction and synchronization of estrous cycle for experimental group (estrous: day 5, metestrous: day 6, diestrous: day 7 and proestrous: day 8) (*P* > .1, *F* = 0.49, ordinary two-way ANOVA; Bonferroni’s Multiple comparison test, *P* > .9 across all data points, N = 7 mice). G, Memory for AA vs EB odor pair was checked one month after the odor pair learning by mice. Memory was similar across both the groups. (*P* > .1, Mann-Whitney test, N = 13 for both groups)

We established that our multimodal learning and memory task can bring about activation of MOB circuitry. Arc immunoreactive cells were observed in the MOB of the whisker intact mice both on TrD 15 and MD 15 (Figure 5A1-A4). To assess if such an activation of olfactory bulb (OB) circuitry can modulate other olfactory driven behaviors, we carried out a non-volatile olfactory discrimination learning and memory tasks in another groups of female mice (Figure 3B). To this end, we carried out go/no-go odor discrimination paradigm^21^ to investigate modulation of discrimination learning and memory of non-pheromonal volatiles in Whitten effect induced female mice (Figure 5B,C). CI vs EU odor pair training was carried out for both the groups before Whitten effect was induced in experimental group to detect any possible bias due to the learning efficacy differences that might be existing between two groups (Figure 5D: CI vs EU ordinary two-way ANOVA, Bonferroni’s multiple comparison test, *P* > .05 for all data points in learning curve). Induction and synchronization of estrous cycle, that is, Whitten effect was achieved in a group of female mice by exposing them directly to male urine and bedding (Figure S8). Once the synchronization was achieved, these mice were trained on an odor-reward association task using the go/no-go odor discrimination paradigm. This was done to observe if MBU exposed mice can achieve quicker learning on an odor-reward association task as compared to mice which were just exposed to their female conspecifics in their cage. We did not find any differences in the learning pace for various odor pairs (Figure 5D: AA vs EB: *P* > .1, *F* = 0.12; BZ vs NN: *P* > .1, *F* = 1.43; HX vs PN: *P* > .1, *F* = 0.19; ordinary two-way ANOVA, Bonferroni’s multiple comparison test, *P* > .05 across all data points for all learning curves). Animals from both groups showed similar motivation levels during the odor discrimination training (Figure 5E, comparison of inter-trial intervals for all odor pairs tested *P* > .05). Individual day accuracies were also plotted for AA vs EB odor pair to compare the day-wise accuracy level changes between the experimental and control groups for 4 continuous days (as the estrous cycle was of 4 days length in synchronized group) during the discrimination training days (Figure 5F: *P* > .1, *F* = 0.49, ordinary two-way ANOVA; Bonferroni’s Multiple comparison test, *P* > .9 across all data points). Discrimination accuracy levels were independent of the estrous stages mice were in. We also checked if the memory for one of the odor pairs (AA vs EB) was enhanced when checked 1 month later after the discrimination training. MBU exposure leading to estrous synchronization did not lead to changes in non-pheromonal volatile discrimination behavior (Figure 5G: *P* > .1, Mann-Whitney test). These results indicate least influence from the estrous stages on the MOB-dependent odor discrimination learning and memory at least with the paradigm we used.

### 5.5 Multimodality involved in pheromone location learning was substantiated by associations involving shape discrimination

**Figure 6:**
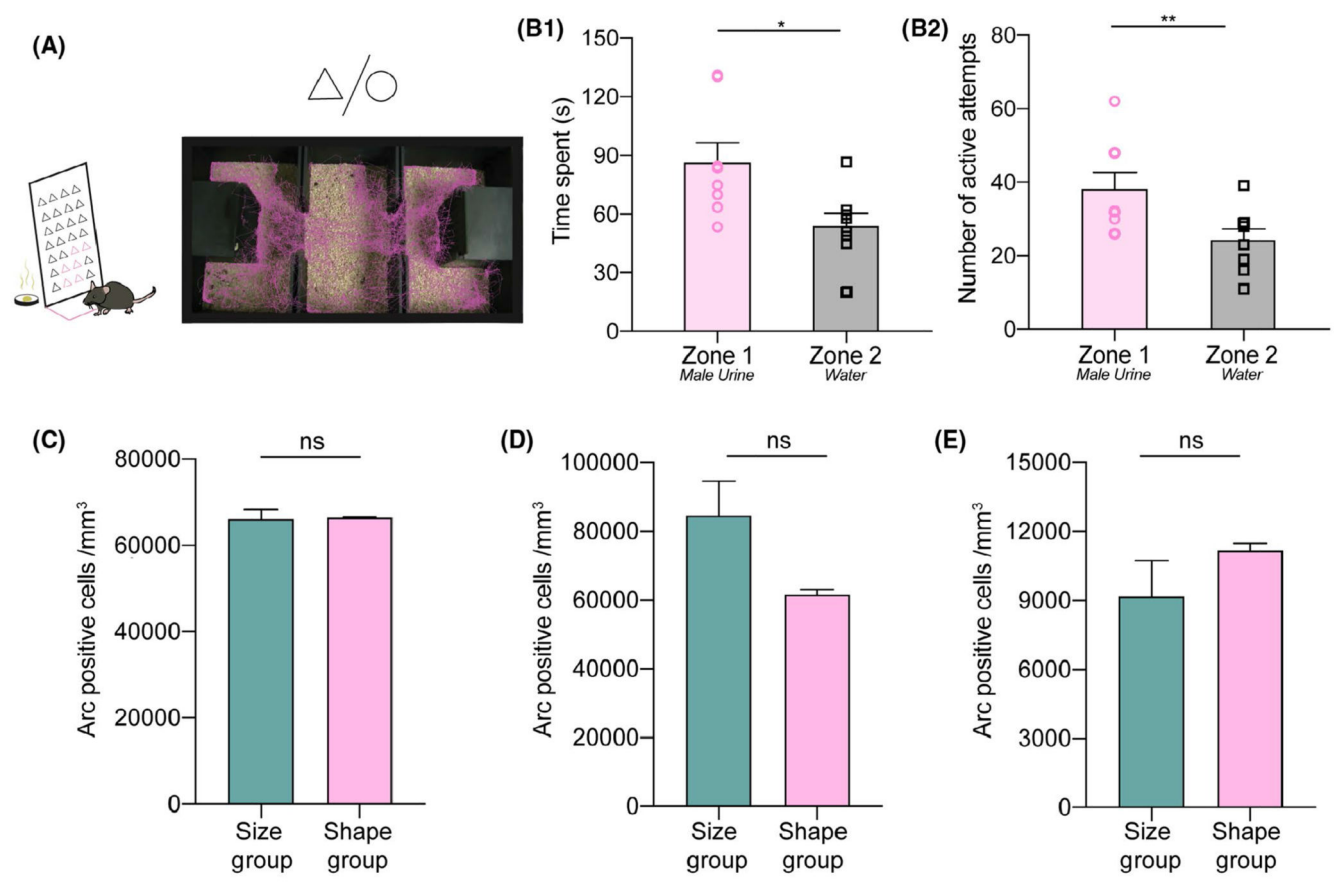
Multimodality involved in pheromone location learning was substantiated by associations involving shape discrimination. A, Schematic representation of a female mouse sensing the volatiles from triangle-shaped orifices of the plate. A track recorded on MD 15 depicting visibly more time spent near zone 1 in a shape based multimodal pheromonal learning paradigm. B1, More time spent in zone 1 on MD 15 when testing for shape based multimodal task (*P* = .011, Paired two-tailed student’s t test, N = 8 mice). B2, Significantly higher number of active attempts toward the plate paired with OSP chamber on MD 15 in a shape based multimodal task (*P* = .004, Paired two-tailed student’s t test, N = 8 mice). C, Similar Arc activation in the MOB of size trained and shape trained groups on MD 15 (*P* = .9, Unpaired two-tailed t test, N = 2-3 mice). D, SSC based evaluation of Arc immunoreactivity between size and shape groups shows similar numbers when checked on MD 15 (*P* = .17, Unpaired two-tailed t test, N = 2-3 mice). E, Hippocampal Arc expression is similar between size and shape groups when checked on MD 15 (*P* = .39, Unpaired two-tailed t test, N = 2-3 mice)

To further validate the multimodal aspect of our paradigm, we also carried out shape-based pheromonal learning in another set of female mice (group 4, see Materials and Methods). To this end, we carried out training entailing mice to associate triangle vs circular orifices (Shape group) with the stimuli present inside the chamber (OSP and NS) (Figure 6A). An increase in time spent and number of active attempts when checked on day 15th after training indicated the presence of shape driven multimodal memory of pheromonal location (Figure 6B1: *P* = .011, paired two-tailed student’s t test, 6B2: *P* = .004, paired two-tailed student’s t test). The coordinated action of sniffing pheromones and whisking through the orifices/triangles leading to memory acquisition and retention can point to the association between olfactory and whisker systems, which led us to investigate the Arc expression in this group of female mice as well. Similar number of Arc positive cells were found in the three brain regions (Figure 6C-E, *P* > .05 for all plots, unpaired t test, two tailed) when compared to the mice who performed the size/diameter-based multimodal pheromonal memory task. Overall, our finding further corroborates to the involvement of associations between multiple sensory systems even while using a shape-based multimodal pheromonal learning paradigm.

## 6 Discussion

Rodents rely mostly on chemical communication for socio-sexual interactions.^26,27^ As these chemical signals can be accompanied with other sensory stimuli, to what extent do they depend on the associations formed between different sensory modalities? Field-based rodent studies have indicated that deposits in the form of urine scent marks act as an interface to showcase an animal’s presence and its ranking in the social hierarchy.^28,29^ Male urine contains releaser pheromones, which can generate attraction in the opposite sex while it can lead to defensiveness and aggression in the same sex.^30–32^ As male mice tend to line their surrounding surfaces with urinary marks,^33^ are olfactory cues alone enough to find and remember the pheromonal locations? In this study, we focused on two quintessential modalities—olfactory and whisker systems in forming associations to facilitate information processing related to pheromone locations.

We investigated this under laboratory setting by creating a training chamber where animals had to use their whiskers while sensing volatile pheromones through orifices of specific sizes and shapes (Figure 1). On training the mice to learn this novel association while gathering information about pheromone locations, we proved the essential role of whisker system in facilitating the same. Our results depict that whisker intact female mice can successfully associate the urinary stimulus with the particular dimensions/shapes of the orifices and remember this association for over a period of 15 days (Figures 1 and 6). The sensory deprivation—whisker trimming and/or blocking of any possible somatosensory cues resulted in the lack of memory formation about pheromone locations. The specificity of learning was shown toward opposite sex urine volatiles compared to signals from the same sex (Figure 2). Neural activation patterns quantified by Arc expressions showed an enhancement during robust memory formation under sensory-intact conditions and a reduction when the memory was lacking under sensory-deprived conditions—hence providing the neural correlates of pheromone location learning and memory (Figures 3 and 4). Despite the enhanced activation patterns in MOB circuit, the olfactory discrimination abilities remained unaffected (Figure 5).

Why would rodents rely on signals from multiple modalities to remember pheromone locations? The environmental background of the natural habitat of rodents is full of volatiles and non-volatile odors.^30,34^ Although non-volatile urinary proteins can act as natural reinforcers for the opposite sex,^35,36^ urine also contains a cocktail of volatile pheromones, which can help in sustaining the attraction of the opposite sex. Indeed, the production of volatile compounds of urine is testosterone dependent and thus, can signal the reproductive potential of the male.^37^ The competence of a female mouse would lie in remembering the specific pheromonal cues in a dynamic and turbulent olfactory space so as to cause successful mating.

The sampling behavior, for example, sniffing plays an important role in making olfactory-guided decisions.^21^ To carry out olfactory/pheromonal-driven exploratory behaviors, we hypothesized the involvement of other sensory modalities that could potentially be in action along with sniffing. Despite the early reports on coordinated action of sniffing and whisking, its functional relevance remains largely elusive.^3^ While respiratory centers in ventral medulla regulate these actions, facial muscles serve these coordinated movements.^2,38–40^ The accuracy to distinguish a target odor from the background decreases as the number of odors in the background mix increases.^41^ The milieu of pheromones displayed to a female mouse is immense and thus, the extent to which the olfactory system can govern distinguishing and remembering specific pheromonal locations is unknown. Therefore, animals might require additional non-olfactory information, which could be the coordinated action of whisking. The potential association of volatile and non-volatile pheromones with the surrounding entities’ size and shape in wild could facilitate the memory formation of pheromone locations in a complex environment.

In our paradigm, the sensation of urinary volatiles from the orifices are occurring simultaneously with the brushing of whiskers and micro-vibrissae along the curvatures of the orifices. Such a phenomenon is important for forming robust association. When whiskers were removed by either just trimming them or by additionally applying lidocaine to reversibly block the sensation by non-mystacial vibrissae, we found out that the mice were unable to learn and memorize the location.^22^ Mice had regrown whiskers when the memory was to be tested 15 days after the training. The sniffing strategies toward the volatile pheromones remained unchanged in whisker deprived mice, as measured by the sniffing frequencies. This ensures that the lack of memory formation we observed under whisker deprived conditions is caused by the reduction in association between two sensory modalities, but not due to alterations in the sampling behavior.

When we trained female mice to associate the female urine and water with the particular orifices, they did not memorize it, when checked 15 days later. This suggests that the extent of learning and thereby the memory formation is decided by weighing of specific cues and the relevance of association formed. Female mice recalled the pheromone location when they were trained for male urine while it was not the case for non-attractive same sex urine even under whisker intact conditions. In multi-sensory associative tasks, different systems might contribute to the final response, to a different extent.^42^ In case of Garcia’s rats, gustatory stimulus was particularly avoided only when the tasty water was paired with a noxious drug. However, it was not affected when a bright-noisy water (an audio-visual stimulus) was conditioned with the noxious stimulus.^43^

Another striking observation we report here is the association formed between shape discrimination (a triangle vs a circle) and pheromone volatiles. Here, different shapes were paired with OSP and NS, in contrast to size discrimination. It is known that whiskers help encoding information of textures, shapes, and even surface angles at a resolution of 15 degrees.^44–47^ In our paradigm, we found that mice can readily memorize pheromone location associating with varying shapes. Mice might be paying more contingency on the attractive pheromonal volatiles when associated with distinct somatosensory stimuli. We propose that whiskers are facilitating the process of associative memory formation, irrespective of the type of somatosensory information collected (shape vs diameter) as deprivation is leading to loss of memory. These observations call for further experiments investigating differential banking of whiskers and vibrissae under different somatosensory stimuli conditions involving shapes, diameters, and textures in complex multimodal tasks associated with olfactory/pheromonal stimuli to probe information integration from these two modalities.

The behavioral results displayed the involvement of olfactory and whisker systems in memorizing the pheromonal location. To look at circuit specific activation and its modulation under different conditions, we investigated the expression of Arc protein in MOB, barrel cortical layers of somatosensory areas and the hippocampus. Arc gene expression is dependent on the extent of neural plasticity achieved under certain behavioral and physiological conditions.^48^ Arc is activated by tasks involving active learning and exposure to novel stimuli.^49^ The spatio-temporal dynamics of Arc expression, compared to other immediate early genes suggests its role in reliably encoding memory traces.^50,51^ Decreased expression of Arc in FBU trained mice as compared to MBU trained ones in MOB, when checked on MD 15 correlates with the absence of memory for SSP. The enhanced Arc activation in MOB could be due to the enhanced recruitment of OB adult-born granule cells (abGCs) in response to the memory formation of pheromone locations. Pheromonal exposure of a dominant male can cause increased cellular proliferation in sub-ventricular zone and increased turnover in MOB.^52^ However, we need to further validate pheromone-dependent multimodal learning and memory induced Arc expression in abGCs for probing their role in pheromonal memory retrieval.

Increased Arc expression in MOB was caused by pheromone location learning in our behavioral paradigm. Indeed urinary volatile components can activate both VNO-AOB and MOE-MOB subsystems.^26,53^ To assess if pheromonal exposure induced MOB activation can modify females’ olfactory learning, we carried out a non-pheromonal odor volatiles go/no-go discrimination task.^16,18,21^ Continuous male urine exposure induced Whitten effect in the female mice.^54,55^ However, the modulation of associative learning by Whitten effect induced neuroendocrine cascades remains largely unexplored. The reports on functional connectivity between MOB and gonadotropin-releasing hormone (GnRH) positive neurons and the indirect effect of dopamine releasing neurons of MOB on olfactory-driven social behaviors tempted us to explore the Whitten effect induced modulations on non-pheromonal olfactory learning and memory.^56–58^ Our study suggests that olfactory learning abilities are unaffected by different estrous stages at least with the go/no-go paradigm we have used. Therefore, these results demonstrate that the probable sex-dependent changes in the behavioral readouts depend primarily on the type of paradigm used and the complexity of the task employed.

Expression of Arc in somatosensory areas upon whisker stimulation and spatial exploration conveys its role in faithfully revealing the specific ensembles activated.^59,60^ Layer 4 of S1 cortex contains “barrels” while the layer 6 contains neurons forming “infrabarrels” which are spatially aligned with barrels of layer 4. Distinctive neuronal types present in layer 6 receive inputs from distinct thalamic sub-regions, thereby, capable of integrating information receiving from different pathways.^61^ We observed a decrease in Arc positive cells in the barrel cortical layers when mice were whisker deprived which is concomitant with decreased number of active attempts on MD 15. In a behavioral task where paired olfactory and whisker stimulations were given, cross-modal reflexes led to enhanced connectivity between olfactory and somatosensory cortices.^62^ Differential Arc immunoreactivity between MD 15 and MD 30 in MOB and SSC correlates with stronger association as seen on MD 15. Remodeling of circuits and connectivity patterns, in the absence of pheromonal exposure, could result in lower Arc activation.

Hippocampus is long known to be a site for activity-dependent plasticity receiving information from multiple sensory modalities, including olfactory and somatosensory regions.^63–66^ DG plays a role in context-dependent discrimination learning and encoding long-term memory.^67^ The decrease in the number of Arc positive cells when the memory is absent on MD 30 depicts its role in the recall. Overall, the multimodal aspect of the pheromone location learning leading to the preference memory formation of urinary pheromones suggests that pheromonal communication is not just a simple stimulus-response system. With respect to socio-sexual preference, female mice could exhibit enhancement of pheromonal location memory when more than one sensory system was mediating the associative learning. Our findings thus provide an evidence for multimodal learning and memory formation in tasks pertinent to social and reproductive behaviors.

## Supplementary Material

Supporting Information

## Abbreviations

AA: amyl acetate
abGCs: adult-born granule cells
AOB: accessory olfactory bulb
Arc: activity-regulated cytoskeleton
BNST: bed nucleus of stria terminalis
BSA: bovine serum albumin
BZ: benzaldehyde
CI: 1,4-cineole
DAPI: 4’,6-diamidino-2-phenylindole
DG: dentate gyrus
DV: diversion valve
EB: ethyl butyrate
EU: eugenol
FBU: female soiled bedding and urine volatiles
GnRH: gonadotropin-releasing hormone
HX: hexanal
IR: infra-red
ITI: inter-trial interval
MBU: male soiled bedding and urine volatiles
MD 15: memory day 15
MD 30: memory day 30
MOB: main olfactory bulb
MOE: main olfactory epithelium
MS4A: membrane-spanning 4-domains subfamily A
NN: nonanol
NS: neutral stimulus
OB: olfactory bulb
OCT: optimal cutting temperature
OSP: opposite sex pheromones
PBS: phosphate-buffered saline
PFA: paraformaldehyde
PN: 2-Pentanone
S-: unrewarded odor
S+: rewarded odor
SSC: somatosensory cortex
TAARs: trace amine-associated receptors
TBS: Tris-buffered saline
TrD 15: training day 15 or last training day
VNO: vomeronasal organ
