# Supplementary material for: Multimodal learning of pheromone locations
Source: FASEB J. Author manuscript; Available in PMC 2021 Oct 13. (PMC7611819; doi:10.1096/fj.202100167R)
Supplement: Supporting Information [file EMS136132-supplement-Supporting_Information.docx]

**Supplemental data**

**Figure S1:**

**
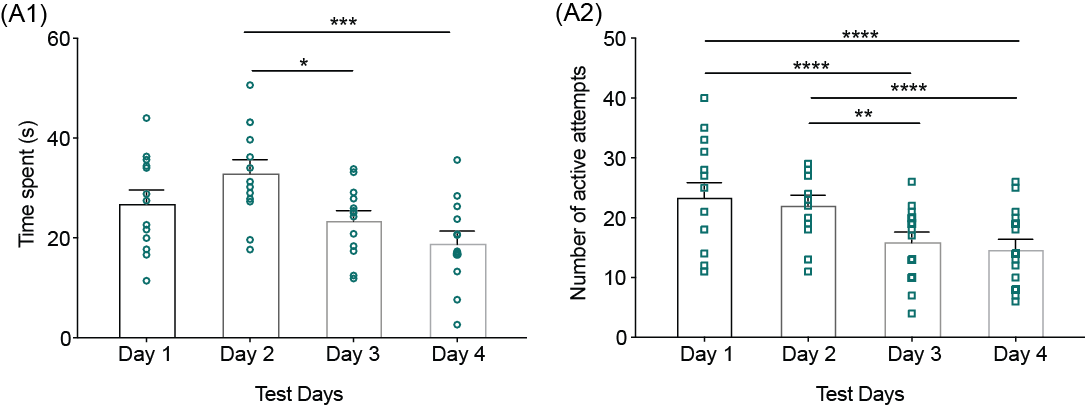
**

**Figure S1. Decayed preference for pheromone locations in whisker-intact female mice exposed to a choice of male urine and neutral stimulus.**

A1. Time spent near OSP chamber containing male urine and soiled bedding by whisker intact female mice. Decayed preference towards this chamber was observed during the initial testing phase (p < 0.01, F = 6.3, RM One-way ANOVA; Bonferroni’s multiple comparison test, p = 0.04 for day 2 v/s day 3 and p = 0.001 for day 2 v/s day 4; p > 0.1 for all other comparisons, N = 13 mice).

A2. Number of active attempts towards OSP chamber (male urine) during the initial 4 days of testing phase. Mice showed lower number of nose pokes on day 3 and 4 (p < 0.0001, F = 16.3, RM One-way ANOVA; Bonferroni’s multiple comparison test, p < 0.0001 for day 1 v/s day 3, 4 and p < 0.001 day 2 v/s day 3, 4; p > 0.9 for all other comparisons, N = 15 mice).

**Figure S2:**

**
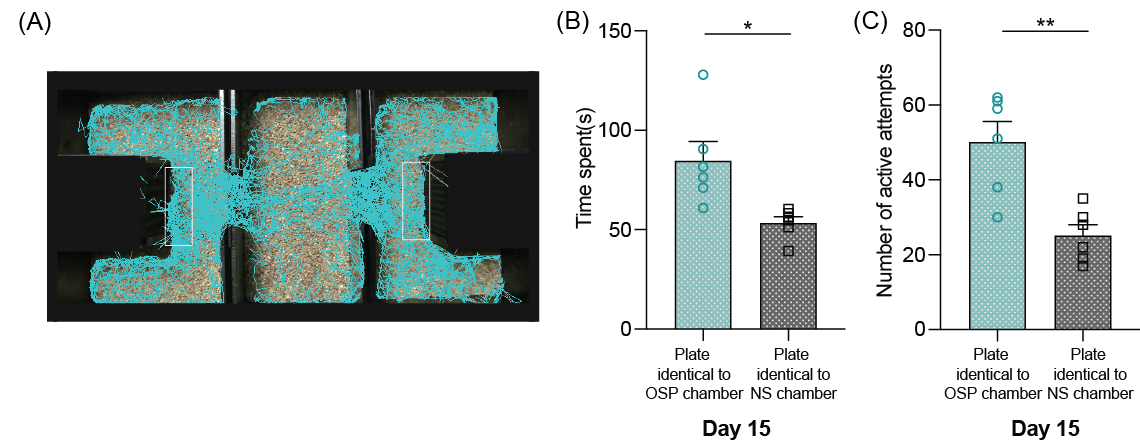
**

**Figure S2. Multimodal Pheromonal memory is not due to the presence of any pheromonal residual cues.**

A. Track recorded in a new, yet identical testing chamber using the ‘nose point’ feature of Noldus EthoVision.

B. Mice spent more time towards a chamber which was guarded by plates which contained orifices of diameters that were identical to the plate used for guarding the OSP chamber during the initial testing and training period (p = 0.01, Paired two-tailed student’s t-test, N = 6 mice).

C. Mice made significantly more number of active attempts into the orifices of the plate which they remembered to be paired with an attractive OSP stimulus during the training period (p = 0.003, Paired two-tailed student’s t-test, N = 6 mice).

**
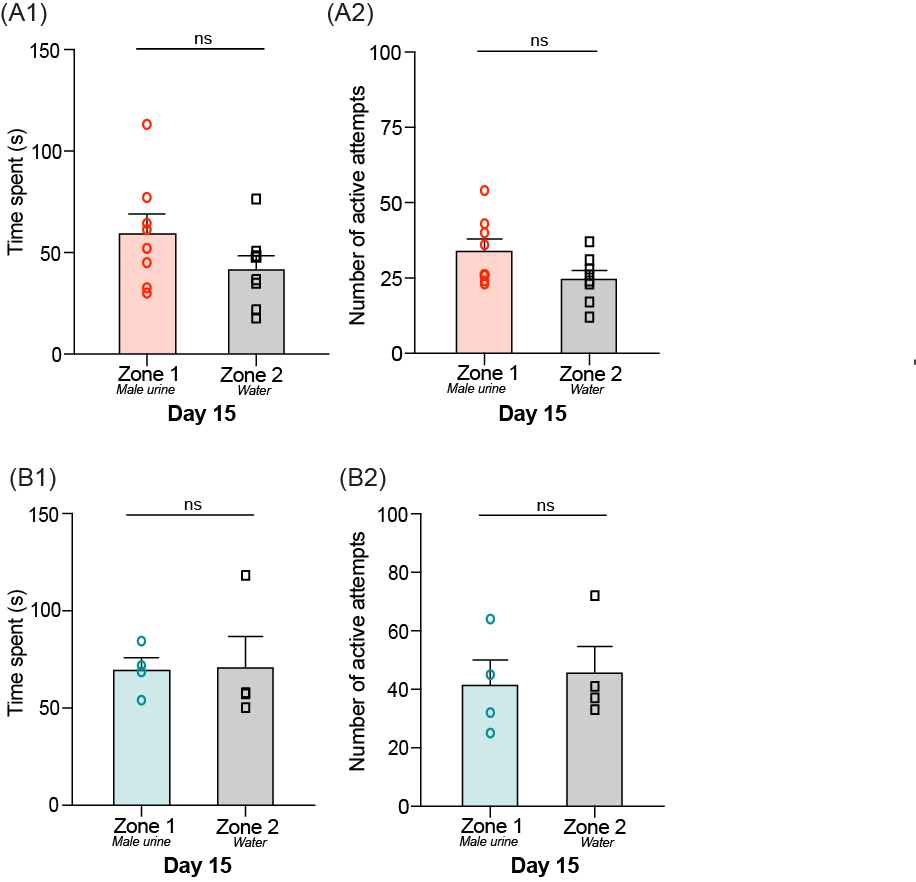
Figure S3:**

**Figure S3. Similar sampling behavior shown towards pheromonal and neutral stimuli in sensory deprived mice.**

A1, A2. No preference for OSP as indicated by similar time spent and number of active attempts for whisker deprived female mice on day 15^th^ memory phase. The mice were trained with male urine and bedding v/s water. (p > 0.1 for time spent and for number of active attempts, Paired two-tailed student’s t-test, N =8 mice).

B1, B2. Whisker trimming alone, unaccompanied with the application of anesthetic gel led to absence of multimodal memory as indicated by similar time spent and number of active attempts for whisker deprived female mice on day 15^th^ memory phase. (p > 0.9, Wilcoxon matched pairs signed rank test, p = 0.71, Paired two-tailed student’s t-test, N = 4 mice).

**Figure S4:**

**
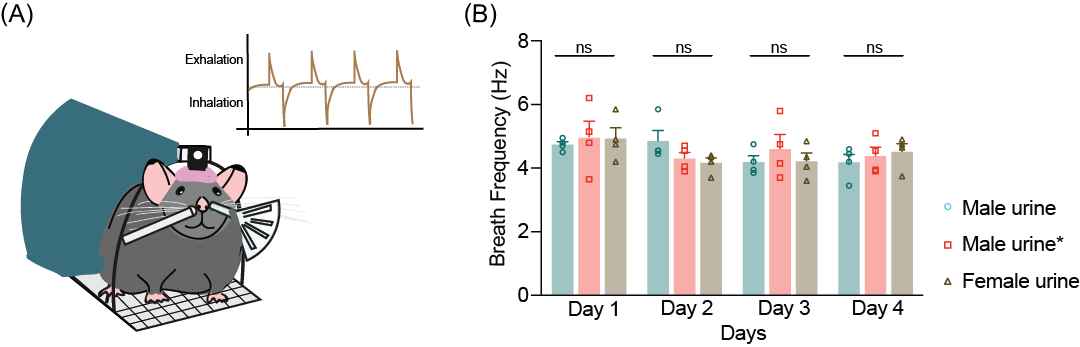
**

**Figure S4. Similar sniffing frequency for sampling pheromones under whisker intact and deprived conditions.**

A. Schematic of a female mouse head-post implanted on the head-restrained set-up. Breathing is recorded using air-pressure sensor placed near one nostril (on left nostril in depiction) and delivering the stimulus (male urine or female urine volatiles) pseudorandomized with filtered air presentations from a nozzle (right side). Inset: Illustration of inhalation/exhalation pattern of a mouse breathing during stimulus delivery.

B. Breath frequency in three groups of female mice (teal: whisker intact sensing male urine volatiles, red: whisker deprived, anesthetic applied sensing male urine volatiles and brown: whisker intact sensing female urine volatiles) recorded across four consecutive days (day 1: p = 0.9, F = 0.1; day 2: p > 0.1, F = 2.2; day 3: p = 0.6, F = 0.50; day 4: p = 0.67, F = 0.40; Ordinary One-way ANOVA, Bonferroni’s multiple comparison test, p > 0.1, N = 4 mice for all groups) (Male urine*: whisker deprived group/Group 2).

**Figure S5:**

**
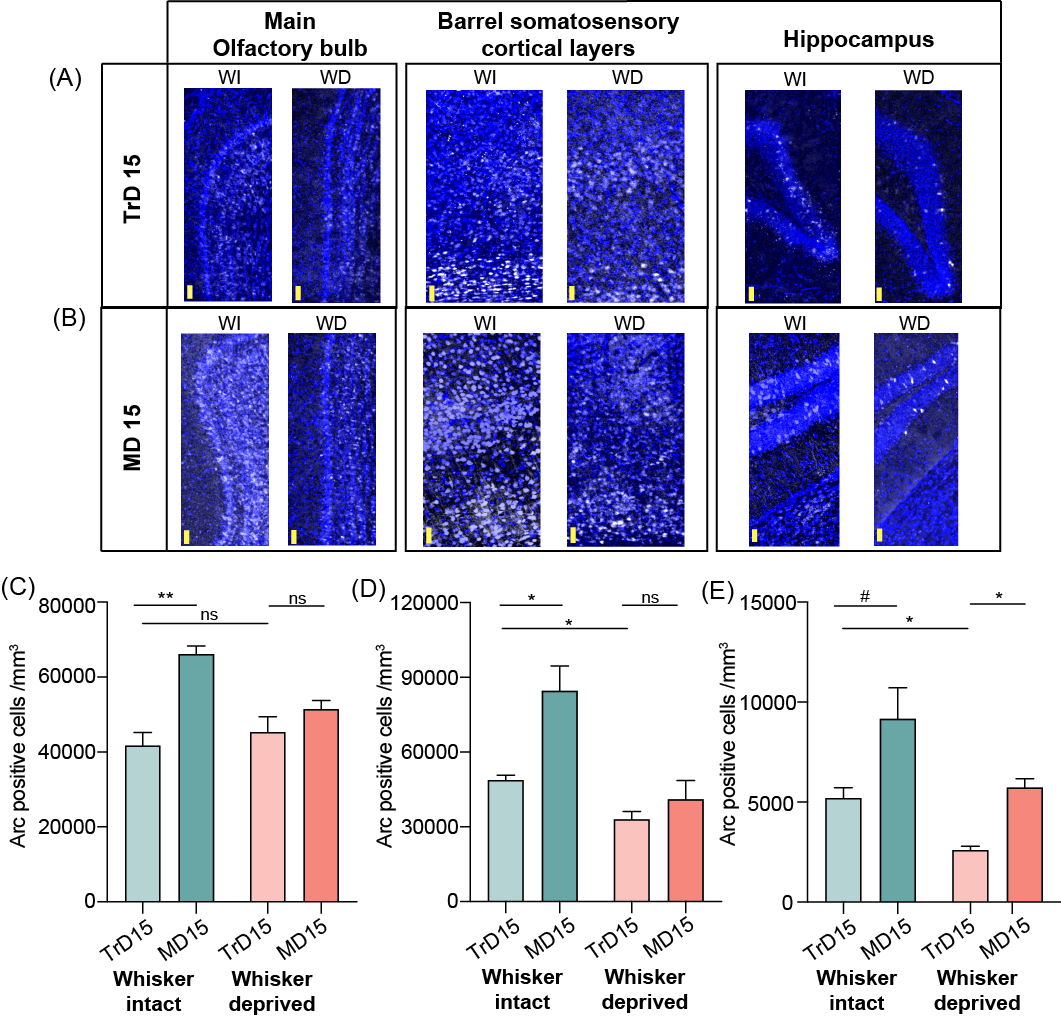
**

**Figure S5.** **Trend of Arc activation during training and memory in the multimodal pheromone learning paradigm in whisker intact and deprived mice.**

A. Immunofluorescence images of Arc immunoreactivity in the MOB, SSC and Hippocampus of whisker intact (WI) and deprived (WD) mice on TrD 15. Blue: DAPI, Gray: Arc, Scale Bar: 50μm.

B. Immunofluorescence images of Arc immunoreactivity in the MOB, SSC and Hippocampus of whisker intact (WI) and deprived (WD) mice on MD 15. Blue: DAPI, Gray: Arc, Scale Bar: 50μm.

C. Increase in MOB activation from last day of training to memory day in whisker intact mice (p = 0.003, Unpaired two-tailed t-test, N = 3 mice), which is not seen in whisker deprived mice (p = 0.24, Unpaired two-tailed t-test, N = 2-3 mice). As MOB sensory activity is not blocked in both the groups during training phase, Arc immunoreactivity is similar in both groups on TrD 15 (p = 0.5, Unpaired two-tailed t-test, N = 3 mice).

D. Increase in Arc positive cells in SSC from training to memory in whisker intact (p = 0.02, Unpaired two-tailed t-test, N = 3 mice) but not in deprived mice (p = 0.48, Unpaired two-tailed t-test, N = 2-3 mice). Even on TrD 15, Arc reactivity is more in whisker intact mice (p = 0.02, Unpaired two-tailed t-test, N = 3 mice).

E. Trend of increase in hippocampal Arc immunoreactivity in both whisker intact (p = 0.07, Unpaired two-tailed t-test, N = 2-3 mice) and increase in whisker deprived mice (p = 0.01, Unpaired two-tailed t-test, N = 3 mice). Particularly on TrD15, Arc positive cells are significantly more in whisker intact mice (p = 0.031, Unpaired two-tailed t-test, N = 3 mice).

**Figure S6:**

**
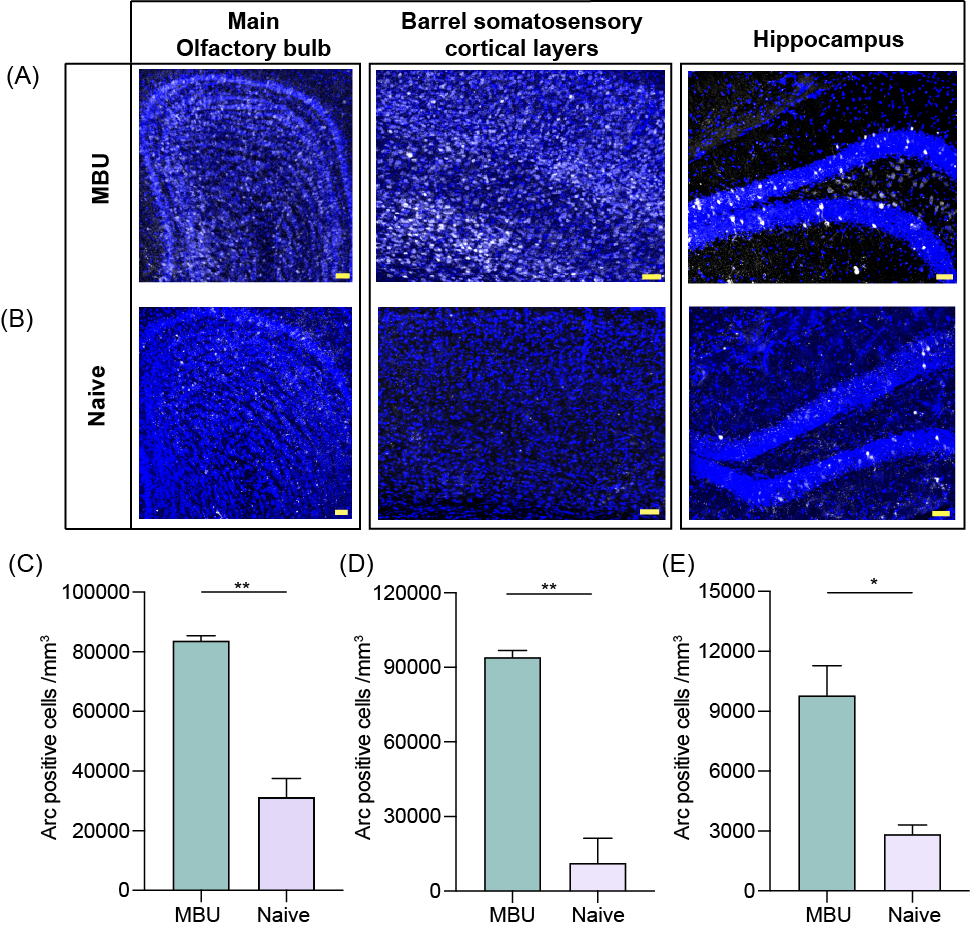
**

**Figure S6.** **Arc expression is specific to male urine and bedding (MBU) based multimodal memory of pheromone locations.**

A. Immunofluorescence images of Arc immunoreactivity in the MOB, SSC and Hippocampus of whisker intact MBU trained & memory tested mice. Blue: DAPI, Gray: Arc, Scale Bar: 50μm.

B. Immunofluorescence images of Arc immunoreactivity in the MOB, SSC and Hippocampus of naïve, singly housed mice. Blue: DAPI, Gray: Arc, Scale Bar: 50μm.

C. Significantly higher number of Arc activated cells in the MOB of mice trained with MBU and tested for the multimodal memory on MD 15 (p = 0.002, Unpaired two-tailed t-test, N = 2-3 mice).

D. Increased Arc activation in SSC barrel cortical layers of MBU trained and tested whisker intact mice when compared to naïve mice (p = 0.002, Unpaired two-tailed t-test, N = 2-3 mice).

E. Increased Arc expression in Hippocampal region of MBU trained and tested whisker intact mice compared to naïve mice (p = 0.03, Unpaired two-tailed t-test, N = 2-3 mice).

**Figure S7:**

**
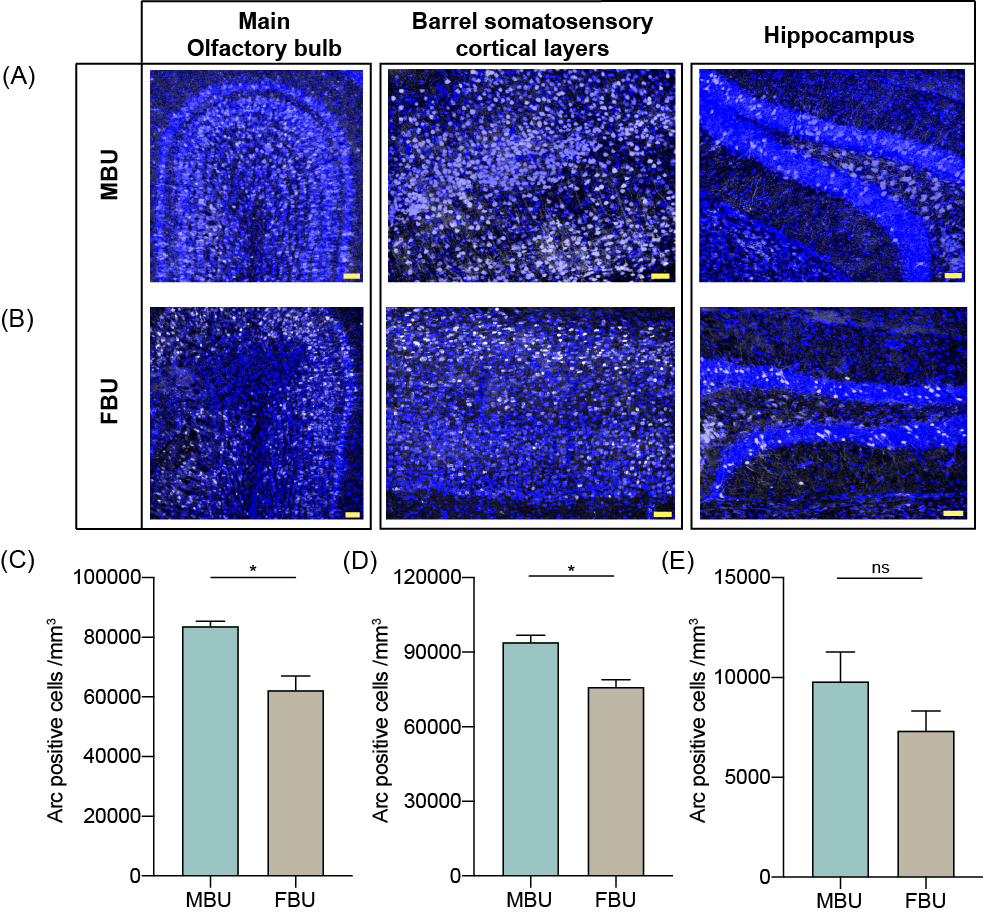
**

**Figure S7.** **Differential Arc expression in MOB and SSC of MBU and FBU trained mice tested for multimodal memory on Memory Day 15^th^.**

A. Immunofluorescence images of Arc immunoreactivity in the MOB, SSC and Hippocampus of whisker intact MBU trained & memory tested mice. Blue: DAPI, Gray: Arc, Scale Bar: 50μm.

B. Immunofluorescence images of Arc immunoreactivity in the MOB, SSC and Hippocampus of whisker intact FBU trained & memory tested mice. Blue: DAPI, Gray: Arc, Scale Bar: 50μm.

C. Lower number of Arc+ cells found in the MOB of female mice trained and tested for FBU in the multimodal learning paradigm (p = 0.013, Unpaired two-tailed t-test, N = 3 mice for each group) depicting robust activation of MOB neurons specific to OSP.

D. Significant reduction in Arc positive cells in the barrel cortical layers of SSC in FBU tested mice (p = 0.011, Unpaired two-tailed t-test, N = 3 mice for each group), which was reflected in reduced active attempts for same sex pheromones compared to opposite sex pheromones. This explains the reduced multimodal pheromone location memory observed for FBU group animals.

E. Comparable number of Arc+ cells in the DG of hippocampal area (p = 0.23, Unpaired two-tailed t-test, N = 3 mice for each group) is suggestive of activation of ensembles occurring as a result of similar exploration.

**Figure S8:**

**
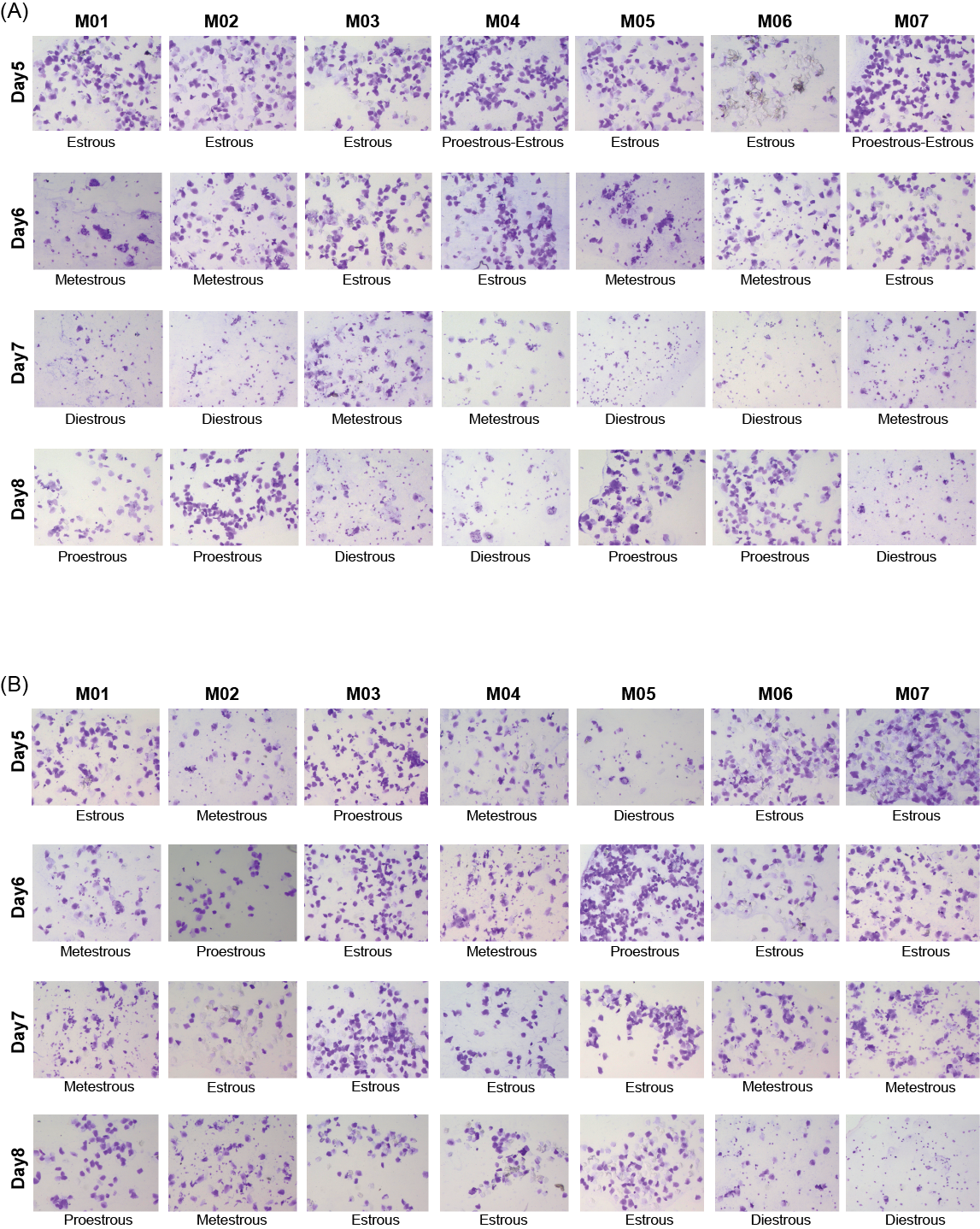
**

**Figure S8. Whitten effect induction and synchronization in female mice by male pheromonal exposure.**

A. Whitten effect, i.e. induction and synchronization of estrous cycle continued to occur in experimental female mice during the duration of AA v/s EB odor pair training. Subset of mice whose vaginal smears were taken on a daily basis during the training, four consecutive days of induction and synchronization is observed in case of experimental mice (each stage lasts for a day, making it a proper 4 day cycle and follows an expected sequence in cycle from proestrous -> estrous -> metestrous -> diestrous).

B. Control group which were not exposed to male urine and soiled bedding exhibited prolonged estrous cycle with one stage lasting > 1 day and not occurring the proper sequence of the cycle, i.e. deviating from proestrous -> estrous -> metestrous -> diestrous sequence.
